# Probing a Reactive Alkyne Center Aligned via a Triphenylmethane Tripod on Au(111) for Electric Field‐Induced Chemistry by STM and TERS

**DOI:** 10.1002/smll.202509467

**Published:** 2026-01-08

**Authors:** Simon Mennicken, Gang Li, Lu‐Yao Zhu, Daniel Schäfer, Till Reichenauer, Saber Mehrparvar, Namhyun Choi, Yao Zhang, Gebhard Haberhauer, Zhen‐Chao Dong, Sebastian Schlücker

**Affiliations:** ^1^ Physical Chemistry I Department of Chemistry and Center of Nanointegration Duisburg‐Essen (CENIDE) University of Duisburg‐Essen 45141 Essen Germany; ^2^ Hefei National Research Center for Physical Sciences at the Microscale and Synergetic Innovation Center of Quantum Information and Quantum Physics and School of Physics and Department of Chemical Physics University of Science and Technology of China Hefei 230026 China; ^3^ Hefei National Laboratory University of Science and Technology of China Hefei 230088 China; ^4^ Organic Chemistry Department of Chemistry University of Duisburg‐Essen 45117 Essen Germany

**Keywords:** molecular orientation, reactive center, rigid tripod, single molecule, tip‐enhanced Raman scattering

## Abstract

Electric field‐induced chemistry at the solid/vacuum interface has distinct advantages over enzyme‐ and electrocatalysis because chemical reactivity is solely governed by external electrical fields (EEFs), while other factors such as solvent and ion effects are ruled out. Intense EEFs on the order of 10^9^ V m^−1^ are generated between the metal tip of a scanning tunneling microscope (STM) and a single‐crystalline metal surface. Reactive centers can be aligned along the direction of the EEF, that is, orthogonal to the metal surface, for experiencing the maximum field. Here, a strategy is introduced to achieve this: a terminal alkyne as a reactive center is connected to the sp^3^‐hybridized core of a triphenylmethane‐based rigid molecular tripod chemisorbed on Au(111), while its upright configuration is probed experimentally by low‐temperature (LT) ultrahigh‐vacuum (UHV) single‐molecule STM and STM‐based tip‐enhanced Raman scattering (TERS) in conjunction with density functional theory (DFT). The orientation of the alkyne moiety relative to the surface normal is inferred from STM height profiles and particularly the Raman intensity of the dominant C≡C stretching peak. The upright configuration and stability of the alkyne reactive center in the presence of intense EEFs paves the way for future E‐field‐induced chemistry monitored in situ by TERS.

## Graphical abstract

Electric‐field‐induced chemistry at solid/vacuum interfaces offers precise control over chemical reactions. In this study, a triphenylmethane‐based tripod with a terminal alkyne is anchored upright on a gold surface. Advanced scanning tunneling microscopy and tip‐enhanced Raman scattering confirm the alignment and stability of the reactive center, enabling new possibilities for electric‐field‐driven molecular reactions.

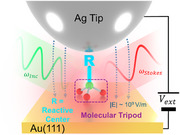

## 1 Introduction

Achieving the highest possible degree of control over chemical reactions is important because it allows scientists and engineers to design processes that are not only effective but sustainable and economically viable. Nature has already achieved this goal in enzymes: they are efficient and highly selective biomolecular catalysts that selectively recognize their substrate and accelerate its selective chemical transformation. Theoretical studies have suggested that oriented external electric fields could be similarly employed to catalyze chemical reactions^[^ ^1^, ^2^, ^3^, ^4^, ^5^, ^6^, ^7^, ^8^, ^9^, ^10^, ^11^, ^12^, ^13^, ^14^, ^15^, ^16^, ^17^, ^18^, ^19^ ^]^ and EEFs have experimentally proven to accelerate a variety of chemical reactions.^[^ ^20^, ^21^, ^22^, ^23^, ^24^, ^25^ ^]^ This emerging field is termed electric field (E‐field) catalysis^[^ ^26^, ^27^, ^28^, ^29^, ^30^, ^31^, ^32^ ^]^ and is distinctly different from the well‐established field of electrocatalysis, in which chemistry occurs due to electron transfer between redox‐active species at electrochemical interfaces, and also different from the catalysis of non‐Faradaic processes at solid‐liquid interfaces.^[^ ^33^ ^]^ This emerging field of E‐field catalysis at the solid‐gas/vacuum interface would have a distinct advantage over enzyme and electrocatalysis in terms of the reduced complexity of the processes involved. Both enzyme catalysis and electrocatalysis are highly complex processes that involve multiple interrelated factors that cannot be disentangled: not only EEFs influence chemical reactivity in these types of catalysis, but processes related to electronic donor/acceptor effects by neighboring groups and quenching of charges by polar solvent molecules at solid/liquid interfaces also play a role. In contrast, chemical reactivity in E‐field catalysis would be solely governed by oriented EEFs, while other factors that cannot be controlled such as solvent and ion effects are excluded. Only recently have researchers begun to experimentally exploit EEFs for accelerating organic reactions. The reason for this temporal delay between theoretical predictions and experimental corroboration is largely due to the associated experimental challenges that must be addressed to realize the promise of E‐field catalysis. The three central challenges are the generation of intense EEFs on the order of 10^9^ V m^−1^ or 1 V nm^−1^, the placement of the reactant molecules in the EEFs in a selectively oriented geometry, and in situ differentiation between reactants and products.

One approach to tackling these challenges has been to use the scanning tunneling microscope‐based break‐junction (STM‐BJ) technique to catalyze chemical reactions. In this approach, the external potential bias of the STM, in combination with the very short distance between tip and substrate, generates the necessary intense oriented EEFs. Product formation is then detected by the change in the tunneling current across the BJ. This approach was used to demonstrate the acceleration of a Diels–Alder reaction using furan as a cyclic, O‐containing and therefore quasi‐nonpolar diene in 2016 experimentally,^[^ ^24^ ^]^ thereby confirming a theoretical prediction by Shaik and co‐workers from 2010.^[^ ^2^ ^]^ This example of an EEF‐catalyzed Diels–Alder reaction with quasi‐nonpolar reactants is a first proof‐of‐concept demonstration of the E‐field catalysis concept.^[^ ^24^ ^]^ A central aspect of that work was the strong immobilization of the dienophile as part of a norbornylogous dithiol bridge via sulfur‐gold bonds to the underlying gold substrate. While this approach oriented the dienophile away from that flat surface, it still suffers from significant conformational flexibility in the sense that the dienophile is not perfectly aligned parallel to the EEF; as a consequence, this starting material does not experience the full strength of the EEF, but only the time‐averaged mean value of its projection. In other words, the full strength of the EEF is not fully exploited for E‐field‐induced chemistry. The same applies to the diene as the second starting material, which was immobilized to the metal tip.

Ideally, the dipole moment associated with the reaction center in the starting material would be aligned perfectly parallel to the external electric field in a defined and rigid manner. In such an orientation, the field can interact most effectively with the dipole, leading to maximal stabilization (or destabilization) of the relevant charge distribution and thereby lowering the activation energy of the reaction. Additionally, it would be desirable to significantly expand the capabilities of the STM by molecular spectroscopy for identifying a broad range of molecules, including conformers. In particular, vibrational Raman scattering offers the advantage of characterizing any type of molecule in situ with chemical specificity.

Multivalent molecular platforms, such as rigid tripodal scaffolds, are a promising approach to achieve a defined and rigid orientation. Applications in combination with metal surfaces and STM range from molecular electronics employing the BJ technique^[^ ^34^ ^]^ to STM‐based electroluminescence.^[^ ^35^, ^36^, ^37^, ^38^ ^]^ Unfortunately, literature reports over the past decade have highlighted major challenges and often contradictory results regarding the orientation of such molecules on metal surfaces. Numerous studies on triphenylmethane‐based tripods have shown that despite the “three‐legged” anchoring motif intended to promote vertical, upright adsorption (“standing‐up”), the actual orientation frequently deviates from the ideal situation. In many cases, STM, X‐ray Photoelectron Spectroscopy (XPS), and Near‐Edge X‐ray Absorption Fine Structure (NEXAFS) studies revealed that flat‐lying or only slightly tilted configurations dominate, especially for meta‐substituted tripods or those with large π‐systems since van der Waals and π–metal interactions with the Au(111) surface often dominate and outperform the geometric preferences imparted by the chelating anchor groups.^[^ ^36^, ^37^, ^38^, ^39^ ^]^ This tendency is particularly pronounced on flat terraces but may be mitigated at step edges or by controlled deposition and annealing protocols.

**Figure 1:**
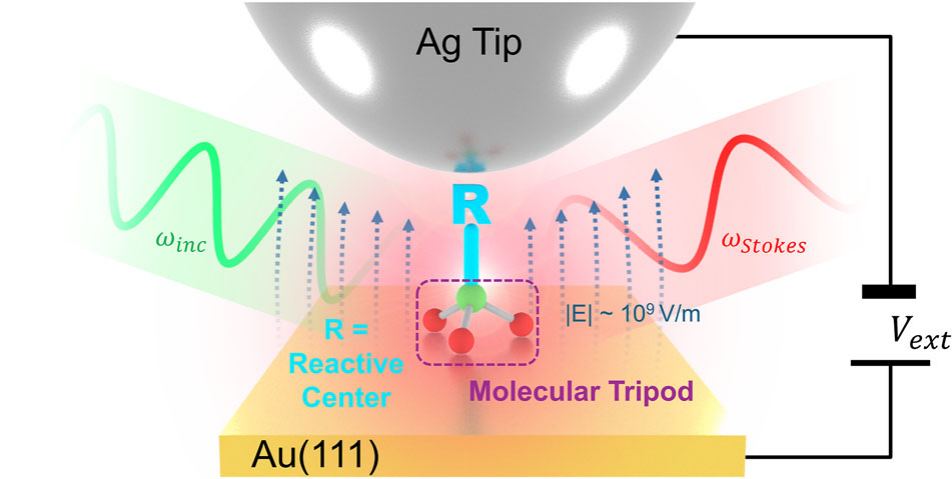
Probing the orientation and stability of a reactive center‐tripodal anchor‐molecule on Au(111) in the presence of strong external electric fields by TERS.

**Figure** **1** schematically depicts our conceptual approach, combining state‐of‐the‐art physical and chemical technologies: scanning tunneling microscopy in the ultrahigh vacuum at low temperatures, single‐molecule plasmon‐enhanced vibrational Raman spectroscopy,^[^ ^40^, ^41^, ^42^, ^43^ ^]^ and a multivalent molecular platform for controlled integration of the reactive center into the EEF. In the present proof of concept, we selected a terminal alkyne as a model system for a reactive center R. The alkyne is well suited for this purpose since it has i) a high intrinsic chemical reactivity (e.g., addition of H_2_ or HX, click chemistry), ii) a high Raman scattering cross section, and iii) the peak of the C≡C stretching vibration occurs in the Raman‐silent region of other components in the molecule and is therefore spectrally separated from the Raman signature of the rigid molecular tripod. STM, STM‐TERS, and DFT are employed for probing the tripod's upright configuration.

## 2 Results and Discussion

**Figure 2:**
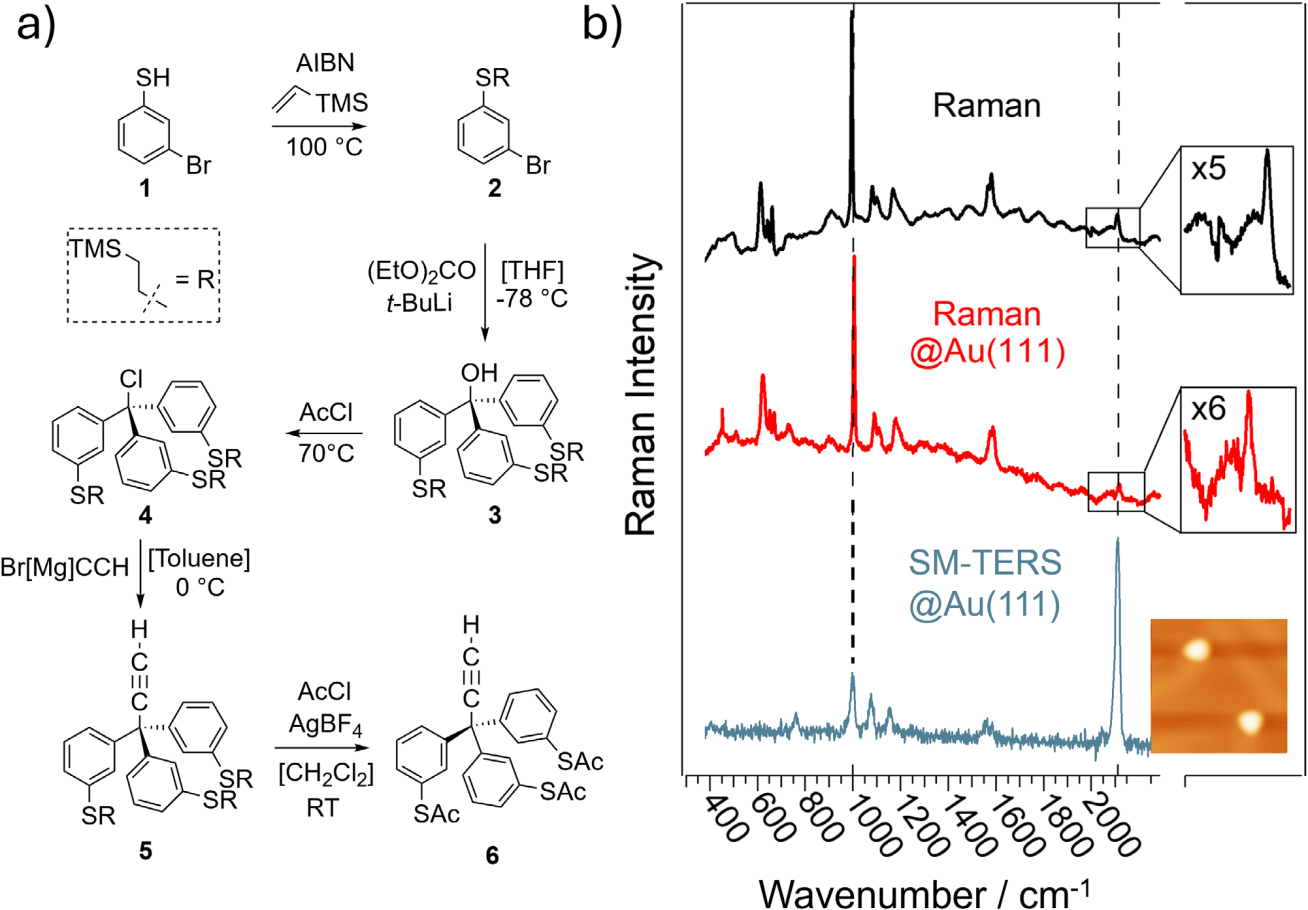
Tripod synthesis and Raman spectroscopic characterization. a) Scheme of the synthesis route to the alkyne‐terminated triphenylmethane‐based tripod **6**. b) Raman spectroscopic characterization of compound **6** (from top to bottom): bulk (black), adsorbed on single‐crystalline Au(111) (red), and single‐molecule TERS recorded on Au(111) (blue). The insets show an enlarged view of the alkyne stretching region and a representative STM image of adsorbed tripod molecules.

**Figure** **2**a shows the scheme for the organic synthesis of the alkyne‐terminated triphenylmethane‐based rigid molecular tripod **6** from the starting material 3‐bromothiophenol (**1**), following the approach from Lindner et al.,^[^ ^39^ ^]^ The thiol group in **1** is protected by a trimethylsilane (TMS) group using a radical reaction with trimethyl(vinyl)silane initiated by azoisobutyronitril (AIBN). The resulting thioether **2** is activated to a C‐nucleophile (3‐position) by the addition of *tert*‐BuLi for the reaction with diethylcarbonate as the electrophile, yielding the trityl alcohol **3**. The latter is converted to the corresponding trityl chloride **4** via activation with acetylchloride. The terminal alkyne in **5** is introduced via the Grignard reagent Br[Mg]CCH.^[^ ^44^ ^]^ Finally, in a one‐pot reaction, the TMS protecting group is cleaved by AgBF_4,_ and the resulting thiol groups are acylated. Both thiol protecting groups, TMS (thioether) and Ac (thioester), prevent homopolymerisation of the trithiols **5** and **6** to the corresponding disulfides. However, in contrast to thioethers, thioesters can easily be cleaved, for example, in solution by the addition of hydroxide.^[^ ^45^, ^46^ ^]^ In this study, the cleavage was achieved by thermal annealing of the Au(111) surface, which was first heated to ca. 150 °C in the preparation chamber and subsequently transferred to the load lock chamber. The solution of **6** in DCM was introduced via pulsed injection about 2 min after heating had stopped, at which point the substrate temperature had typically decreased to ca. 80 °C. Figure 2b depicts the Raman spectra of the alkyne‐terminated rigid molecular tripod **6**: as a neat compound (top) and after adsorption on Au(111) (middle). The LT‐UHV TERS spectrum of a single molecule is also shown (bottom). Both non‐plasmon‐enhanced Raman spectra (top and middle) are dominated by the intense ring breathing vibration of the three phenyl rings at ca. 1003 cm^−1^. The C≡C stretching vibration of the alkyne moiety at ca 2130 cm^−1^ in the Raman‐silent region of other components in the molecule is enlarged in the corresponding insets. Both characteristic Raman peaks are also observable in the single‐molecule LT‐UHV TERS spectrum (bottom). However, in contrast to the two non‐plasmon‐enhanced Raman spectra, the C≡C stretching peak clearly dominates in the TERS spectrum. We attribute this selective enhancement of the C≡C stretching peak to both position and, in particular, orientation of the alkyne moiety in the plasmonic nanogap. Specifically, the terminal C≡C bond is close to the tip and aligned parallel to the surface normal, that is, parallel to the vector of the localized electric field in the hot spot. The STM image in the bottom inset shows two molecules located on the Au(111) surface.

The TERS spectrum was recorded from the molecule in the top left corner. (For more correlated STM TERS data, we refer the reader to Figure S1, Supporting Information).

**Figure 3:**
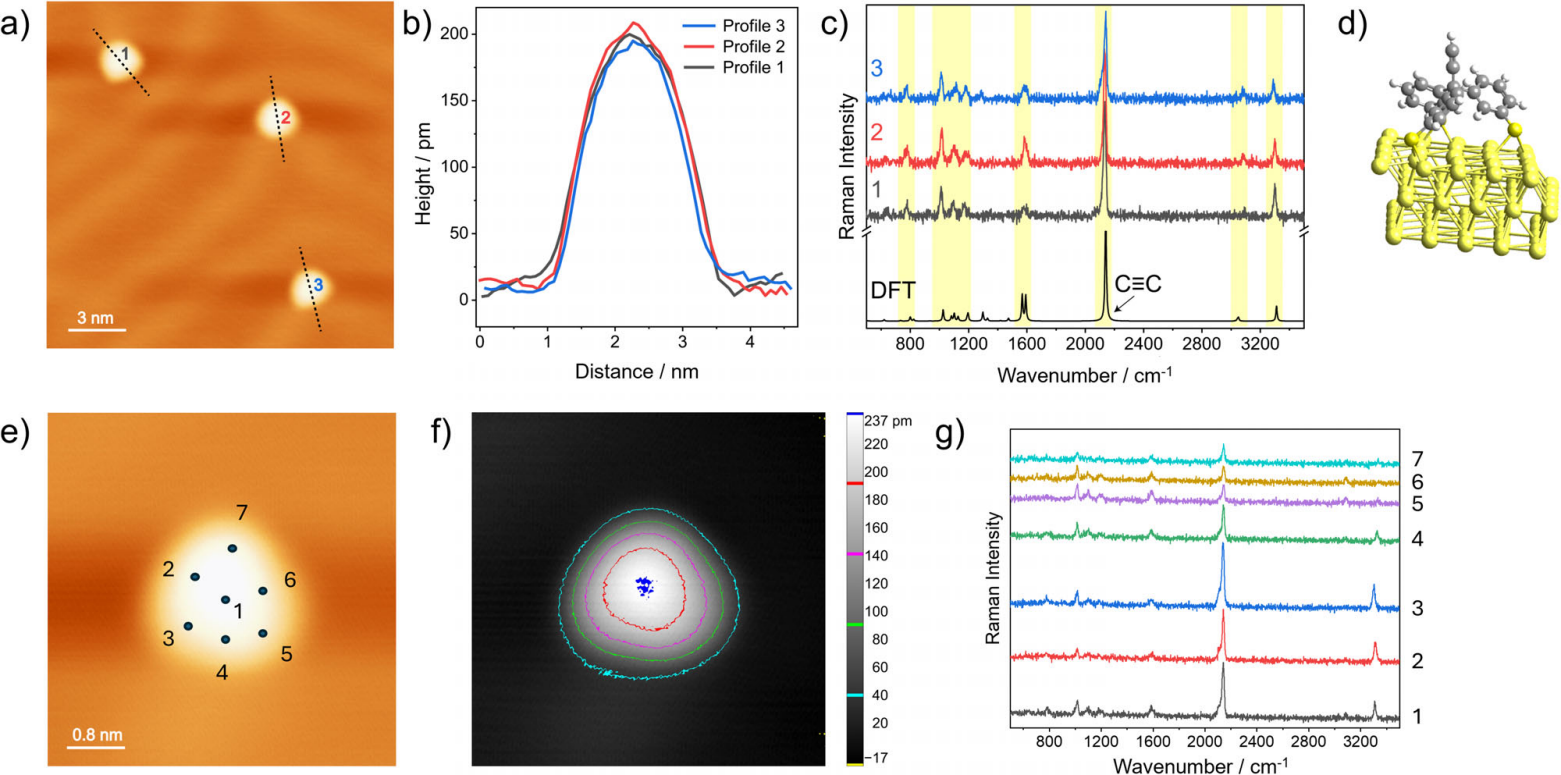
Upright configuration of the tripod on Au(111) terraces. a) STM image of three individual alkyne‐terminated tripod molecules adsorbed on terraces of the Au(111) surface (−1 V, 5 pA). b) Corresponding apparent height profiles. c) Corresponding experimental and single‐molecule TERS spectra (−0.1 V, 2 nA, 10 s). Also plotted at the bottom is the DFT‐simulated TERS spectrum for the upright configuration. d) Schematic of the upright configuration. e) STM image (−1 V, 5 pA) of a single alkyne‐terminated tripod molecule together with the seven positions of a “radial” TERS mapping experiment. f) Corresponding STM‐derived false‐color height image. g) Corresponding seven TERS spectra (−0.1 V, 500 pA, 10 s).

We aimed to find out whether alkyne‑terminated tripod molecules adsorbed on terraces of the Au(111) surface reproducibly adopt an upright configuration. We also examined their exact orientation relative to the surface normal. **Figure** **3**a shows an STM image with three single tripod molecules adsorbed on terraces of the Au(111) surface. The herringbone pattern is clearly visible. The corresponding height profiles are depicted in Figure 3b. All three tripods exhibit an apparent height of ca. 200 pm. In the corresponding experimental single‐molecule TERS spectra in Figure 3c, the characteristic Raman peaks of the triphenylmethane‐based core (fingerprint region), the dominant C≡C stretching peak (≈2126 cm^−1^), and the alkyne C─H stretching peaks (high‐wavenumber region) are clearly visible. The agreement with the theoretical Raman spectrum (Figure 3c bottom) of the upright configuration in Figure 3d is very good, as indicated by the yellow bars for selected Raman peaks. In the optimized geometry (Figure 3d), the terminal alkyne has an inclination of 15° relative to the surface normal, indicating a non‐perfect orthogonal orientation. In order to experimentally probe this, we performed a TERS mapping experiment with seven positions distributed radially around a single tripod molecule (Figure 3e). The corresponding 2D height profile is depicted in Figure 3f, where different colors represent different heights above the surface. The highest point is located near the center of the molecule, consistent with an upright orientation of the tripod. This spatial topography provides a direct visualization of the single tripod on Au(111) and enables precise assignment of the TERS measurement positions around the molecule. In the corresponding TERS spectra (Figure 3g), the intensity of the dominant C≡C stretching peak as well as of the alkyne C─H stretching peak differs substantially. The maximum Raman intensity is observed at position 3, followed by positions 1 and 2 with roughly equal intensities. At position 4 ca 50% of the maximum intensity is observed. In contrast, the Raman intensities at positions 5 to 7 are less than ca. 25% of the maximum intensity. The minimum intensity occurs at position 6, that is, opposite to position 3. We attribute this intensity pattern to an orientation of the terminal alkyne toward position 3. To exclude the possibility that the observed intensity differences of the alkyne vibrational peaks are caused by a non‐isometric tip generating locally stronger plasmonic fields due to surface irregularities, we measured two molecules consecutively under identical conditions. In these experiments (see Figure S2, Supporting Information), we observed the same intensity differences at random positions, supporting our hypothesis that the alkyne group is slightly tilted in this direction.

The experimental STM and TERS data as well as the computed results in Figure 3 strongly support an upright configuration of alkyne‐terminated tripods on Au(111) terraces. The same behavior is also observed for many other single tripod molecules. However, this observation differs from previous findings reported in the literature. Instances of upright adsorption have been reported for related systems under specific conditions, although the degree of orientational order and stability varies with molecular design and deposition method.^[^ ^35^, ^36^, ^37^, ^38^ ^]^ For example, Zhu et al. demonstrated that a self‑decoupled porphyrin with a rigid tripodal anchor can adopt an upright configuration on Au(111), enabling efficient coupling of the transition dipole to nanocavity plasmons and resulting in strong electroluminescence.^[^ ^35^ ^]^ Likewise, hot‑luminescent NDI chromophores mounted on extended tripodal scaffolds^[^ ^36^ ^]^ and rigid tripod–chromophore conjugates of varying core substitution^[^ ^37^, ^38^ ^]^ have shown that upright arrangements — when achieved — lead to enhanced optical activity and reduced quenching. In contrast, several studies on other rigid tripodal systems, including meta‑substituted tetraphenylmethane tripods^[^ ^39^ ^]^ and conjugates with bulky chromophores,^[^ ^36^, ^37^, ^38^ ^]^ report a predominant flat‑lying geometry stabilised by extended π–surface interactions, with upright species representing the exception rather than the rule, especially for bulkier or more π‑extended head groups. For example, in the case of meta‑ and para‑thiol‑substituted tetraphenylmethane tripods, UHV‑STM and NEXAFS data suggest a dominance of flat‑lying configurations in islands, while upright orientation is observed only in specific environments such as at step edges (meta isomers) or under multilayer formation (para isomers).^[^ ^39^ ^]^ Related NDI‑based tripods tend to form unordered horizontal assemblies, with upright configurations occasionally induced by tip manipulation.^[^ ^38^ ^]^ Taken together, the comparison with Refs. [35, 36, 37, 38, 39] indicates that achieving upright adsorption is highly system‑dependent and requires a combination of rigid anchoring geometry, suitable head‑group size, and carefully chosen deposition conditions.

**Figure 4:**
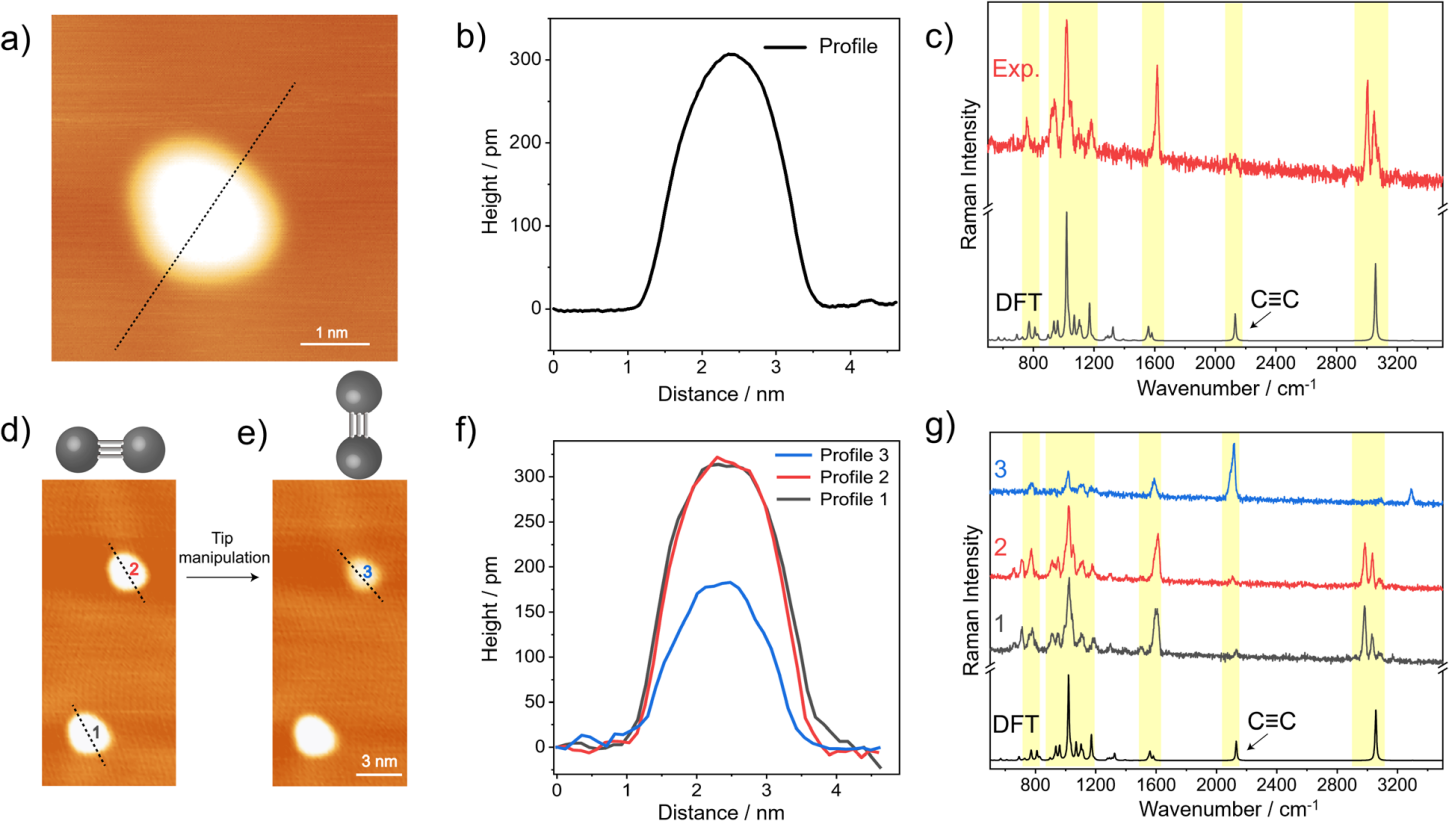
Rare flat‐lying configuration (top) and tip manipulation (bottom). a) STM image of a single alkyne‐terminated tripod molecule adsorbed on a Au(111) terrace (−1 V, 2 pA). b) Corresponding apparent height profile. c) Corresponding experimental single‐molecule TERS spectra (−0.1 V, 500 pA, 10 s), together with the theoretically predicted Raman spectrum for a flat‐lying configuration. d,e) STM images (−1 V, 2 pA) before (d) and after (e) tip manipulation, which was performed by approaching the tip closer to the molecule at a tunneling condition of −0.1 V and 300 pA, and line‐scanning across the molecule. f) Corresponding apparent height profiles. g) Corresponding experimental single‐molecule TERS spectra.

Although upright tripod molecules are by far the dominant species, rare cases with a flat‐lying configuration on Au(111) terraces are observed. **Figure** **4**a shows a STM image of a single alkyne‐terminated tripod molecule on a Au(111) terrace, for which both the corresponding height profile in Figure 4b and particularly the corresponding TERS spectrum in Figure 4c suggest an orientation in which the terminal alkyne group is oriented nearly parallel to the surface. The apparent height is ca. 300 pm, which is ca. 50% larger with respect to the case of the upright standing molecules (b). We attribute this to the larger size of the phenyl ring compared to the alkyne moiety, which is pointing up in this case. The weak intensity of the alkyne stretching band near 2126 cm^−1^ is a clear indicator for a flat orientation because this vibrational mode does not experience a TERS enhancement along the direction of the tip. This is supported by the DFT‐calculated Raman spectrum shown below (for the optimized geometry, we refer the reader to Figure S3, Supporting Information). Interestingly, a single alkyne‐terminated tripod molecule can be actively switched from a flat‐lying to an upright configuration via tip manipulation. The STM image in Figure 4d displays two molecules, for which both the corresponding STM height profiles (Figure 4f, apparent height ca. 300 pm) and the corresponding TERS spectra (Figure 4g, weak alkyne C≡C stretching peak) clearly indicate a flat‐lying configuration. After tip manipulation of molecule **2**, its brightness in the STM image in Figure 4e is reduced compared to that of molecule **1**. Both the corresponding height profile 3 in Figure 4f (apparent height ca. 200 pm) and the corresponding TERS spectrum (Figure 4g, intense alkyne C≡C stretching peak) clearly indicate an upright configuration for molecule **3**. This suggests a successful reorientation from a flat lying to the energetically more stable upright form on Au(111) terraces. (We note that the switching from the upright to the flat‐lying configuration is also possible, and can be realized by approaching the tip to the molecular edge at a tunneling condition of −0.1 V and 1 nA, though statistically with a success rate of ≈30%.)

**Figure 5:**
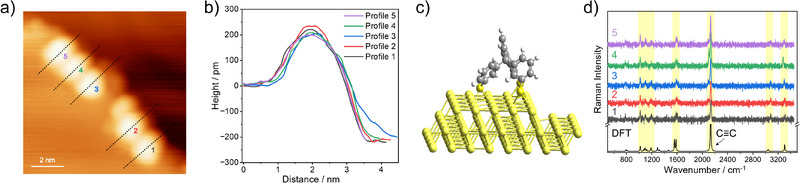
Upright configuration of the tripod at monoatomic step edges. a) STM image showing five individual tripod molecules (−1 V, 5 pA). b) Corresponding apparent height profiles. c) Theoretically predicted adsorption geometry. d) Corresponding experimental single‐molecule TERS spectra (−0.1 V, 1 nA, 10 s), together with the theoretically predicted Raman spectrum at the bottom.

In addition to terraces, the Au(111) surfaces also contain monoatomic step edges, and therefore, the question arises whether the tripods exhibit an upright configuration in this case, too. **Figure** **5**a displays an STM image containing such a monoatomic step edge. All five alkyne‐terminated tripod molecules are located right at this monoatomic step edge. The corresponding height profiles in Figure 5b show an apparent height of ca. 200 pm, and the corresponding single‐molecule TERS spectra in Figure 5d exhibit an intense alkyne C≡C stretching peak, strongly supporting an upright configuration. This conclusion is supported by results from DFT calculations. The energetically most stable adsorption configuration of the tripod shown in Figure 5c comprises two sulfur atoms located directly at the monoatomic step edge. Thus, the latter provides a unique binding environment, resulting in an upright configuration leading to an intense alkyne C≡C stretching peak in the theoretically predicted Raman spectrum. The step edge likely facilitates all three sulfur anchor groups binding to undercoordinated gold atoms, stabilizing the upright configuration and minimizing steric hindrance between the molecule and the gold substrate. This observation is further supported by earlier literature reports. For example, Lindner et al. found in UHV‐STM studies that meta‐substituted tetraphenylmethane tripods often line up along step edges and that these sites can promote alternative adsorption geometries compared to terraces.^[^ ^39^ ^]^ Their analysis suggests that the increased number of available low‐coordination sites at the edge enhances anchoring flexibility and can favor the vertical orientation of multipodal molecules. In other words, while the flat terrace supports an equilibrium between the energetically favored upright configuration on the one hand and the less stable, rarely occurring flat‐lying orientation on the other hand, the monoatomic step edge seems to selectively stabilize the upright configuration (for a broader STM scan including more molecules we refer the reader to Figure S6, Supporting Information). This finding directly corroborates earlier findings^[^ ^39^ ^]^ and highlights the critical role of the local surface environment in governing the adsorption configuration of tripods on single‐crystalline gold surfaces.

**Figure 6:**
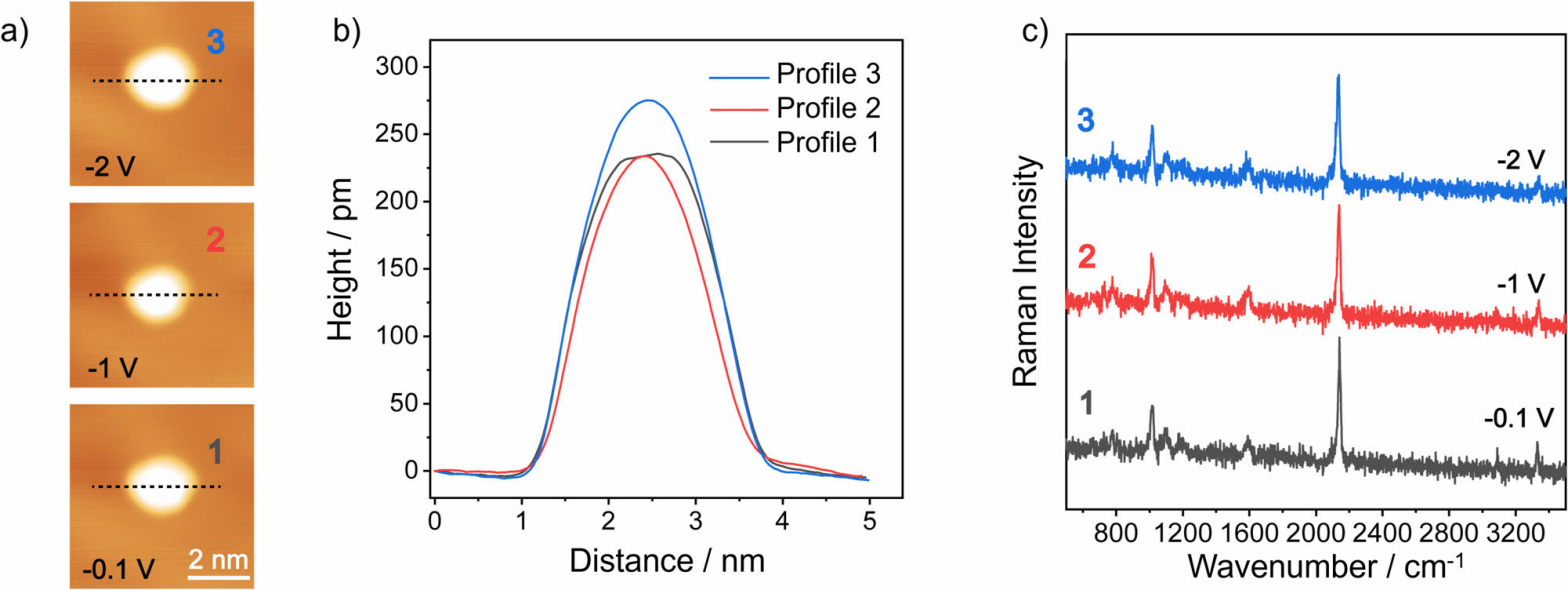
Tripod stability in the presence of intense EEFs. a) STM constant‐current images (5 pA) of the same molecule recorded at sample biases of −0.1 V, −1.0 V, and −2.0 V. The latter, together with an estimated tip‐sample distance of ca. 600 pm, corresponds to an EEF of ca. 3 × 10⁹ V m^−1^. b) Corresponding apparent height profiles. c) Corresponding single‐molecule TERS spectra acquired in the constant‐height mode with a spectral integration time of 10 s.

In addition to an upright configuration, the stability of the alkyne‐terminated tripod in the presence of intense EEFs is a second prerequisite for the investigation of electric field‐induced chemistry. Even under such conditions, the reactive molecular center must remain geometrically fixed and chemically intact. We therefore experimentally tested the robustness of alkyne‐terminated tripods adsorbed on Au(111) terraces since it is the most frequently encountered situation. **Figure** **6** shows the results obtained for a single alkyne‐terminated tripod on a Au(111) terrace that was sequentially exposed to an increasing negative external potential bias with the tip‐sample distance being held constant. The working hypothesis was that any tip‐ or bias‐induced displacement or deformation and change in the adsorption geometry is detectable in the corresponding topographic and vibrational spectroscopic data, that is, via the STM images and height profiles as well as especially via the TERS spectra as a sensitive probe of molecular orientation. Figure 6a displays three consecutive STM images of the same molecule, recorded with a fixed tunneling current of 5 pA but varying sample biases of −0.1 V, −1.0 V, and −2.0 V. Both the central position and the circular appearance of the alkyne‐terminated tripod remain unchanged across the entire bias range, indicating a robust adsorption geometry and the molecule's mechanical stability on the gold surface. This conclusion is corroborated by the corresponding height profiles in Figure 6b and the corresponding TERS spectra in Figure 6c: the unchanged apparent height of 200 pm, as well as the unchanged intense alkyne C≡C stretching peak, are both characteristic for an unchanged upright configuration. The applied electric field strengths can be calculated from the estimated tip–sample distance of ca. 600 pm and the corresponding applied voltages; they range from ca. 2 × 10⁸ V m^−1^ at −0.1 V to 3 × 10⁹ V m^−1^ at −2.0 V. The latter leads to a vibrational Stark shift of ca. 4 cm^−1^ for the alkyne C≡C stretching peak.

The stability of the alkyne‐terminated triphenylmethane‐based tripod under intense EEFs demonstrates that the underlying rigid molecular architecture offers a stable and well‐defined molecular platform for aligning reactive centers under such extreme conditions. This exquisite robustness in the presence of EEFs is a crucial prerequisite for future investigation of electric field‐induced chemistry that can be monitored in situ by TERS.

## 3 Conclusion

Through covalent bonding of a terminal alkyne to a rigid triphenylmethane‐based molecular tripod in combination with sample preparation comprising pulsed injection, we have achieved precise control over the orientation of the reactive alkyne center with respect to intense EEFs in a plasmonic nanogap. The dominant upright configuration of tripod‐supported alkyne conjugates on Au(111) terraces and monoatomic step edges was corroborated by correlative single‐molecule low‐temperature UHV–STM and TERS experiments. Especially TERS as a vibrational spectroscopic technique contributed the highly relevant information on the orientation.^[^ ^47^, ^48^ ^]^ In this case, the orientation of the single reactive alkyne center is unambiguously determined, independent from the size and shape of the supporting tripod; achieving this by STM alone is limited in general. In addition, we found that rarely observed flat‐lying tripods can be switched to the more stable upright configuration by tip manipulation. Furthermore, tripod‐supported alkyne conjugates are found to maintain their orientation and chemical stability even in the presence of intense EEFs on the order of few 10⁹ V m^−1^. Our approach presents state‐of‐the‐art chemical and physical technologies to E‐field‐induced chemistry: rational design of reactive center‐rigid tripod conjugates, optimized sample preparation for achieving an upright configuration of reactive centers, and experimental characterization by correlative single‐molecule STM/TERS measurements. The versatility of TERS for monitoring not only starting material but also reaction products will be exploited in future work on E‐field‐induced chemistry.

## 4 Experimental Section

All chemicals were of reagent grade and used without further purification unless otherwise stated. Solvents were dried and purified following standard literature procedures where necessary. Reactions were monitored by thin‐layer chromatography (TLC) on silica gel 60 F254 plates and visualized under ultraviolet light (254 and 366 nm) or by staining with appropriate reagents (e.g., KMnO_4_) when applicable. Flash column chromatography was performed on silica gel 60 (40–63 µm, 230–400 mesh) using appropriate solvent systems as indicated in the respective experimental sections.

^1^H and ^13^C NMR spectra were acquired on a Bruker Avance DRX 400 MHz spectrometer at ambient temperature. Chemical shifts (*δ*) are given in parts per million (ppm) relative to the residual solvent peaks (e.g., CDCl_3_: *δ*_H_ = 7.26 ppm, *δ*_C_ = 77.0 ppm). Coupling constants (*J*) are reported in Hertz (Hz). All ^13^C NMR spectra were recorded with broadband ^1^H decoupling. Multiplicities are indicated as s (singlet), d (doublet), t (triplet), q (quartet) or m (multiplet). High‐resolution mass spectra (HR‐MS) were obtained on a Bruker BioTOF III mass spectrometer equipped with an electrospray ionization (ESI) source and operated in positive or negative ion mode as indicated. Samples were introduced by direct infusion or via a syringe pump. Mass calibration was performed using a standard calibration mixture to ensure accurate mass determination within ±5 ppm.

1‐Bromo‐3‐[(2‐(trimethylsilyl)ethyl)thio]benzene (**2**)

In a 50 mL pressure tube, 4‐bromothiophenol (**1**) (10.73 g, 56.6 mmol), vinyltrimethylsilane (6.70 g, 9.75 mL, 66.6 mmol), and azobisisobutyronitrile (AIBN) (493 mg, 3 mmol) were added under an argon atmosphere. The tube was sealed, and the mixture was stirred at 100 °C for a duration of 16 h. Upon cooling to room temperature, the solution was diluted with hexane (100 mL) and passed through a short column of silica gel (12 g, hexane). After evaporation of the solvent, the residue was subjected to purification by medium‐pressure chromatography on silica gel (24 g) using pure hexane. This process yielded product **2** as a colorless oil, with a 91% yield (R_f_ = 0.26 in hexane).

For **2**: **^1^H NMR** (400 MHz, CDCl_3_) *δ* = 7.41 (m, 1H), 7.28 (m, 1H), 7.20 (m, 1H), 7.12 (m, 1H), 2.98 – 2.93 (m, 2H), 0.95 – 0.91 (m, 2H), 0.05 ppm (s, 9H). ^13^C NMR (101 MHz, CDCl_3_) *δ* = 140.1, 130.9, 130.2, 128.6, 127.1, 122.9, 29.4, 16.8, −1.6 ppm.

Hydroxytris(3‐[(2‐(trimethylsilyl)ethyl)thio]phenyl)methane (**3**)

A dry, argon‐flushed 100 mL Schlenk flask was charged with **2** (0.982 g, 3.39 mmol), and anhydrous THF (10 mL) was added. The solution was cooled to −78 °C, and *tert*‐butyllithium (4 mL, 6.8 mmol, 1.7 M in pentane) was added dropwise over 10 min. The reaction mixture was stirred at −78 °C for 2 h under argon, followed by the slow addition of diethylcarbonate (0.115 mL, 0.97 mmol). After 1 h, the reaction mixture was allowed to warm to room temperature and stirred for an additional 16 h. The reaction mixture was quenched with saturated NH_4_Cl solution (40 mL). The aqueous layer was washed with dichloromethane (2 × 40 mL). The combined organic layers were washed with brine (40 mL), dried over anhydrous Na_2_SO_4_, and filtered. All volatiles were removed under reduced pressure. The residue was purified by medium‐pressure liquid chromatography on high‐performance silica gel (12 g) using hexane/ethyl acetate (9:1), yielding pure compound **3** as a white solid, with a yield of 53%. $(R_{f}=0.44,\;\mathrm{hexane}/\mathrm{EtOAc}\;=\;9:1)$ For **3**: **^1^H NMR** (400 MHz, CDCl_3_) *δ* = 7.29–7.26 (m, 9H), 7.09–7.07 (m, 3H), 2.95 – 2.91 (m, 6H), 2.84 (s, 1H), 0.95 – 0.91 (m, 6H), 0.07 ppm (s, 27H). **^13^C NMR** (101 MHz, DMSO‐d_6_) *δ* = 147.1, 137.4, 128.5, 127.9, 127.5, 125.4, 81.8, 29.3, 16.8, –1.6 ppm. **HRMS (ESI‐TOF)** m/z: [C_34_H_52_OS_3_Si_3_+Na]^+^ calculated: 679.2380; found: 679.2375.

Ethynyltris(3‐[(2‐(trimethylsilyl)ethyl)thio]phenyl)methane (**5**)

In a dry, argon‐flushed Schlenk flask, **3** (73 mg, 0.110 mmol) was suspended in acetyl chloride (1.1 mL, 15.4 mmol), and the reaction mixture was stirred for 60 min at 75 °C and then for 14 h at 50 °C. After evaporation of the volatiles, the oily crude product **4** was redissolved in dry toluene (5.0 mL), followed by the addition of ethynylmagnesium bromide (0.22 mL, 0.110 mmol, 0.5 M in THF, Merck) at room temperature. The mixture was stirred at 80 °C overnight. The reaction was quenched with water (10.0 mL), and the aqueous phase was extracted with ethyl acetate (3 × 20.0 mL). The combined organic layers were washed with brine and dried over Na_2_SO_4_. Purification by medium‐pressure chromatography on silica gel using hexane/ethyl acetate (3:1) gave 42 mg (57%) of **5** (R_f_ = 0.72, hexane/EtOAc = 9:1) as a colourless oil. For **5**: **^1^H NMR** (400 MHz, CDCl_3_) *δ* = 7.23–7.17 (m, 9H), 7.00–6.97 (m, 3H), 2.88–2.83 (m, 6H), 2.70 (s, 1H), 0.86 (m, 6H), 0.00 ppm (s, 27H). **^13^C NMR** (101 MHz, CDCl_3_) *δ* = 145.0, 137.5, 129.0, 128.5, 127.3, 126.4, 89.1, 74.1, 55.5, 29.3, 16.8, –1.6 ppm. **HRMS** (ESI‐TOF) m/z: [C_36_H_52_S_3_Si_3_+H]^+^ calculated: 665.2612; found: 665.2602. [C_36_H_52_S_3_Si_3_+Na]^+^ calculated: 687.2431; found: 687.2424.

Ethynyltris(3‐(thioacetyl)phenyl)methane (**6**)

A dry, argon‐flushed 100 mL Schlenk flask was loaded with **5** (0.12 g, 0.16 mmol), AgBF_4_ (0.22 g, 1.12 mmol), and acetyl chloride (1.2 mL) dissolved in dichloromethane (12 mL). The reaction was allowed to proceed at an ambient temperature for 12 h. Subsequently, the reaction mixture was subjected to purification by medium‐pressure chromatography on silica gel (6 g), using hexane/ethyl acetate (3:1). This process yielded 0.07 g of compound **6** (R_f_ = 0.36, hexane/EtOAc = 3:1) as an orange solid, achieving an 81% yield. For **6**: **^1^H NMR** (400 MHz, CDCl_3_) *δ* = 7.39–7.32 (m, 9H), 7.31–7.26 (m, 3H), 2.78 (s, 1H), 2.38 ppm (s, 9H). **^13^C NMR** (101 MHz, CDCl_3_) *δ* = 193.7, 145.0, 135.0, 133.5, 130.1, 129.2, 128.3, 88.2, 75.1, 55.2, 30.3 ppm. **HRMS** (ESI‐TOF) m/z: [C_27_H_22_O_3_S_3_+Na]^+^ calculated: 513.0623; found: 513.0626.

### Sample Preparation for Conventional, Non‐Plasmon‐Enhanced Raman Spectroscopy

Raman measurements of free ethynyltris(3‐(thioacetyl)phenyl)methane (**6**) molecules were carried out without solvent by applying the oil to a slide. Au(111) single crystals were purchased from Surface Preparation Laboratory SPL (Zaandam, The Netherlands) and were used without further surface preparation. First, the acetyl protecting group was cleaved with a 100 mM sodium methanolate solution in THF in a 1:1 ratio with a 1 mM solution of the tripod in THF. 20 µL of the deprotected solution of Tripod in THF was pipetted onto the crystal using the DropCast method. The crystal was washed 3 times with THF to remove unbound tripod molecules and NaOMe residues.

### Raman Instrumentation

The Raman spectra were obtained using a home‐built confocal Raman microscope comprising an inverted microscope (Nikon Eclipse Ti–S) and a grating spectrometer (HORIBA iHR550, 600 grooves mm^−1^ grating) equipped with a back‐illuminated CCD (HORIBA Synapse). The 632.8 nm radiation from a He–Ne laser was focused onto the sample using a 60× microscope objective (Nikon, NA 0.8). The laser power at the sample was 5.5 mW. Acquisition parameters for the tripod on Au(111): 5 accumulations, each 30 sec. 11).

### TERS Sample Preparation

The tripod‐alkyne molecules were dissolved in tetrahydrofuran (≥ 99.9%, Sigma–Aldrich) at a concentration of 100 µM to prepare the solution. Au(111) surfaces were prepared from gold films with a thickness of ≈150 nm via electron beam evaporation of gold onto the cleaved mica in vacuum. The Au(111) surface was first cleaned by cycles of argon ion sputtering and annealing, and then the tripod‐alkyne molecules were deposited on the clean Au(111) surface by using the oblique pulse injection method at room temperature, described in detail in previous works.^[^ ^49^ ^]^ Specifically, the solution was injected into the high‐vacuum chamber through a pulse valve (Parker Corporation) at a slanting angle of ≈45°onto the Au(111) substrate. The Au(111) substrate was heated to ≈150 °C before spraying, and the distance between the pulse valve and the substrate surface was 160 mm. The solution was injected toward the substrate when the pulse valve was opened for 1.5 ms.

### STM and TERS Measurements

All STM imaging and TERS measurements were performed on a custom low‐temperature ultrahigh‐vacuum (LT‐UHV) STM in a confocal‐type side‐illumination configuration at ≈80 K under a base pressure of ≈1×10^−10^ Torr. More experimental details can be found in our previous report.^[^ ^43^ ^]^ Electrochemically etched silver tips were used, which were cleaned by electron‐bombarding and argon‐ion sputtering under UHV and followed by further atomistic modifications through gentle tip indentation onto a clean Ag(110) surface to achieve TERS‐active status for subsequent TERS measurements on Au(111). Tip cleanness is confirmed by taking TERS measurements on the bare metal surface that show featureless spectra above 200 cm^−1^. A single‐longitude‐mode diode‐pumped laser at 532 nm (Changchun New Industries Optoelectronics Tech. Co., Ltd, China) with a free‐space output was used for Raman excitation. A round, continuously variable metallic neutral density filter was used to adjust the laser output power. Two reflective mirrors were used to provide freedom for optical alignment. A half‐wave plate was used to achieve the desired *p*‐polarization for the incident laser. The laser beam was introduced into the UHV chamber via a quartz viewport after being reflected by a 10:90 (R:T) beam splitter. Another beam splitter, which was removed during Raman measurements, was positioned in the optical collection path to help monitor the focusing of the laser beam into the tunnel junction with a video camera. The collimated beam was re‐focused by an aspheric lens (f = 19.5 mm, NA = 0.5) onto the tip−substrate junction at an angle of 60° from the surface normal. The diameter of the laser spot on the sample surface at optimum focus was ∼30 µm. Raman scattered light was collected through the same lens, filtered to remove residual laser light, and then fiber‐coupled to a spectrometer using a slit size of 100 µm. The Raman signal light was dispersed by a 600 or 1200 grooves mm^−1^ grating and detected by a liquid‐nitrogen cooled charge‐coupled‐device (CCD) (Princeton Instruments). The laser power used in the TERS experiments was ≈400 µW before introduction into the UHV chamber. The STM operation was controlled by Nanonis‐SPECS electronics. All STM imaging measurements presented here were carried out in the constant‐current mode. The thermal drift was ≈13 pm min^−1^ laterally and ≈10 pm min^−1^ vertically.

### Quantum‐Chemical Calculations

Quantum‐chemical calculations were performed using the CP2K software^[^ ^50^ ^]^ for periodic structure optimization and the Gaussian 16 suite^[^ ^51^ ^]^ for subsequent cluster‐based analyses. The adsorption complex, comprising the alkyne‐terminated tripod adsorbed on a two‐layer (each containing 64 atoms) Au(111) substrate, was geometry‐optimized via CP2K under periodic boundary conditions using the PBE exchange–correlation functional. Double‐zeta plus polarization quality molecularly optimized basis sets (MOLOPT‐SR‐DZVP)^[^ ^52^ ^]^ and norm‐conserving Goedecker–Teter–Hutter (GTH)^[^ ^53^, ^54^, ^55^ ^]^ pseudopotentials were used. Long‐range dispersion effects were included via Grimme's DFT‐D3 scheme with Becke–Johnson damping (DFT‐D3(BJ)). The kinetic energy cutoff was 300 Ry, and the cutoff of the reference grid 40 Ry. Brillouin‐zone sampling was performed on a Monkhorst–Pack grid of 2 × 2 × 1 k‐points.

Subsequently, the molecule and 9 Au surface atoms in direct contact were extracted to form a cluster model. Here, it is assumed that the substrate has been optimized to a reasonably stable configuration, and the consideration of only those surface metal atoms that are bonded to the molecule can effectively mimic the influence of the infinite substrate. This cluster underwent geometry optimization followed by vibrational frequency analysis using Gaussian 16, with gold atoms constrained to their positions. The calculations employed the B3LYP functional with the 6–311++G(d,p) basis set for C, H, S, and N atoms, and the Stuttgart/Dresden (SDD) effective core potential for Au atoms. Dispersion interactions were included using Grimme's D3 correction with Becke–Johnson damping.^[^ ^56^ ^]^ To facilitate comparison with experimental spectra, the calculated vibrational frequencies were scaled by a factor of 0.9305 followed by application of a +100 cm^−1^ wavenumber shift. The Raman spectra are simulated under external light illumination by taking into account the Raman selection rule.^[^ ^43^, ^57^ ^]^

## Conflict of Interest

The authors declare no conflict of interest.

## Supporting information



Supporting Information

## Data Availability

The data that support the findings of this study are available from the corresponding author upon reasonable request.
