# Supplementary material for: Probing a Reactive Alkyne Center Aligned via a Triphenylmethane Tripod on Au(111) for Electric Field‐Induced Chemistry by STM and TERS
Source: Small. 2026 Jan 8;22(7):e09467. doi: 10.1002/smll.202509467 (PMC12862450; doi:10.1002/smll.202509467)
Supplement: Supplementary file 1 — Supporting Information [file SMLL-22-e09467-s001.docx]

Supporting Information

**Probing a reactive alkyne center aligned via a triphenylmethane tripod on Au(111) for electric field-induced chemistry by STM and TERS**

Simon Mennicken^‡^, Gang Li^‡^, Lu-Yao Zhu, Daniel Schäfer, Till Reichenauer, Saber Mehrparvar, Namhyun Choi, Yao Zhang, Gebhard Haberhauer, Zhen-Chao Dong*, Sebastian Schlücker*

^‡^These authors contributed equally

*Corresponding authors

# Figures


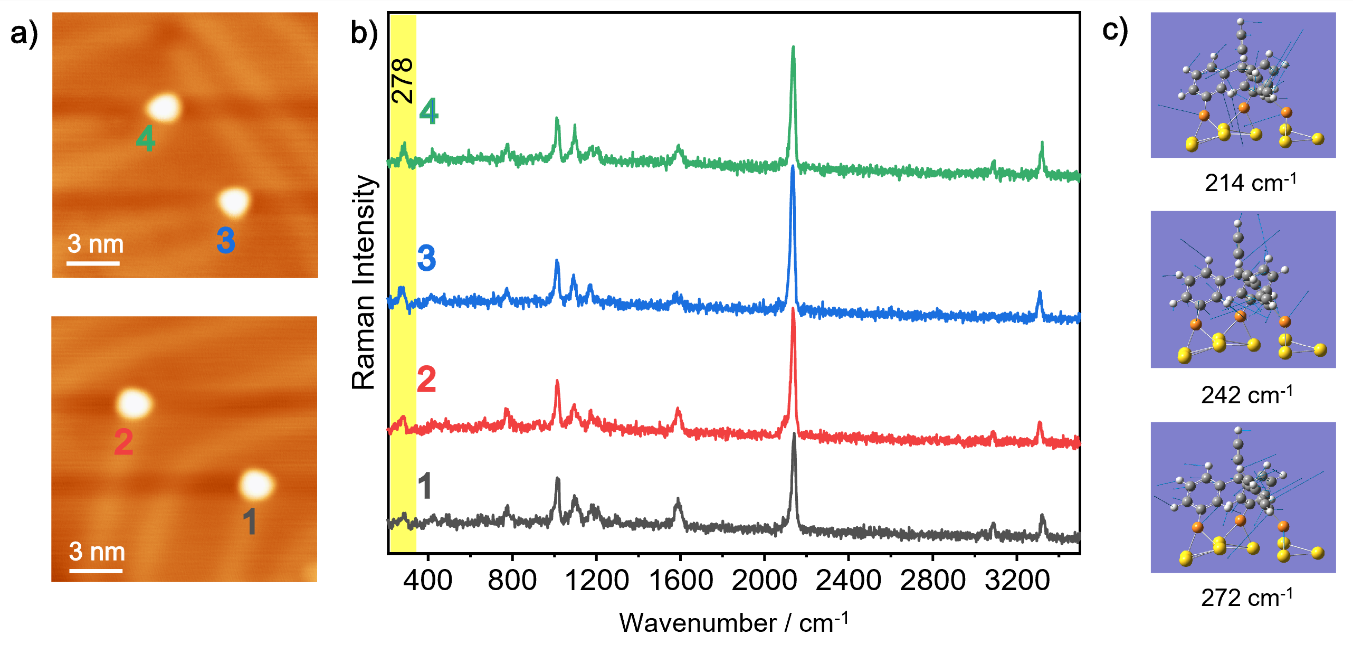


**Figure S1**: a) STM images (−1 V, 5 pA) of four single molecules (upright) with the same height on a Au(111) terrace. b) Corresponding TERS spectra of these four molecules (−0.1 V, 1 nA, 10 s). We would like to note that a small peak consistently appears around 278 cm⁻¹ in the TERS spectra measured above the triphenylmethane-based rigid tripod molecules. This peak can be assigned to the Au-S vibrational mode based on the quantum-chemical simulations shown in sub-panel c) as well as its consistency with the peak position reported in the SERS literature^[1]^ , which clearly indicates the cleavage of the thioester groups upon the deposition of the molecule onto the Au(111) surface. c) eigenvectors of three Au-S vibrational modes and the corresponding wavenumbers.


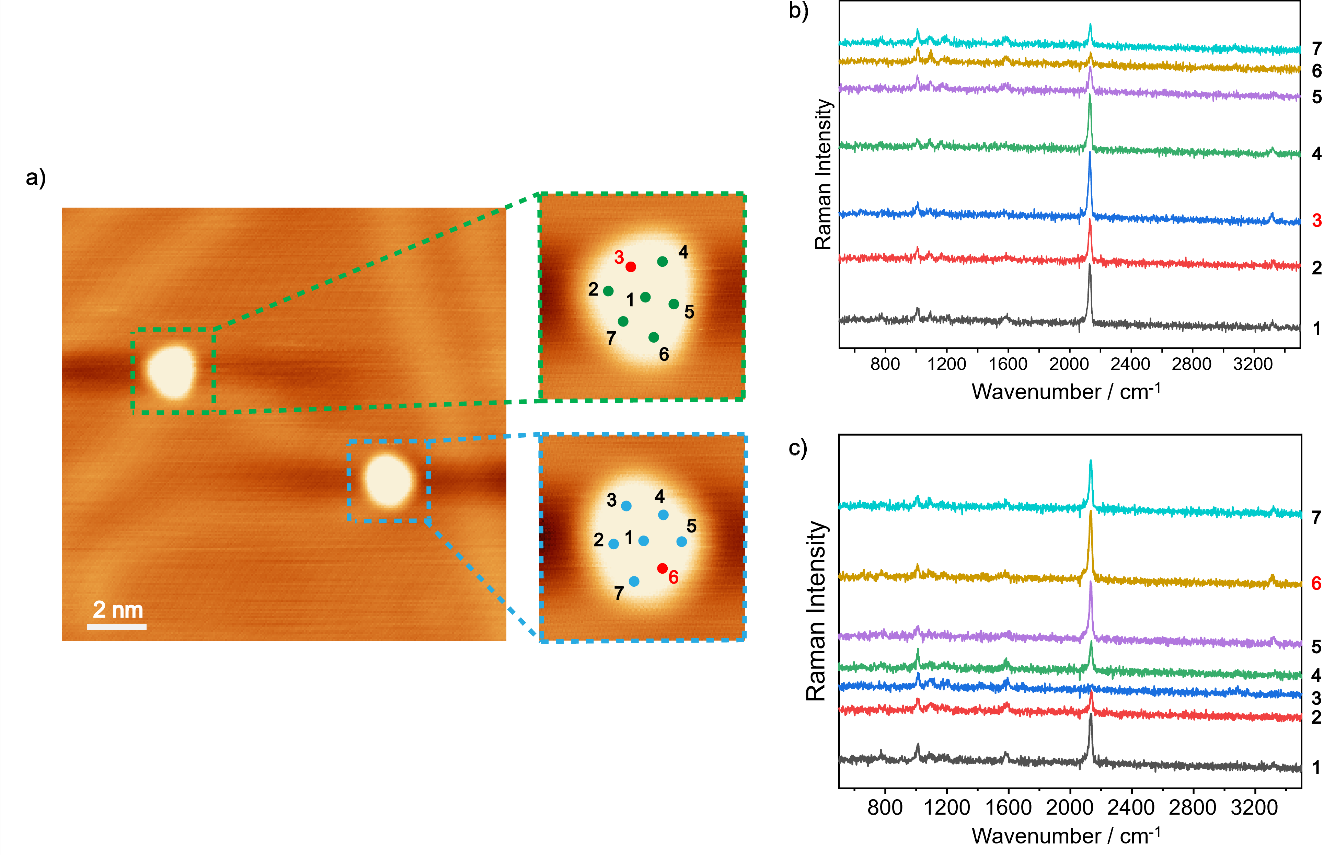


**Figure S2**. Intramolecular TERS mapping (7 positions) for two molecules, which suggest different orientations of the terminal alkyne. a) STM images (−1 V, 5 pA), b) and c) Corresponding TERS spectra of these two molecules with different intensities of the alkyne stretching peak (−0.1 V, 500 pA, 10 s).


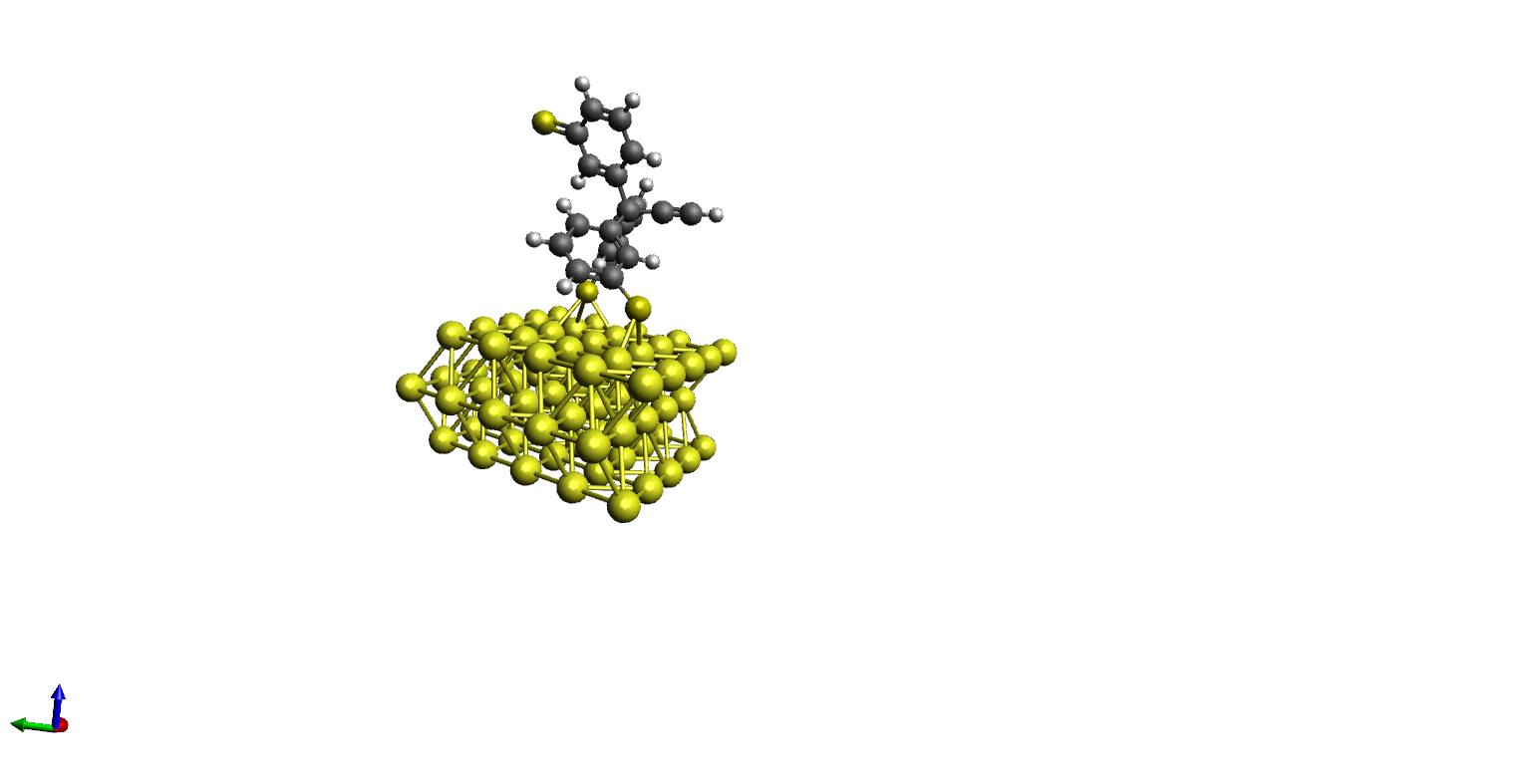


**Figure S3**. Optimized structure of a flat-lying alkyne-terminated tripod with two sulfur atoms binding to gold atoms of the underlying Au(111) surface.


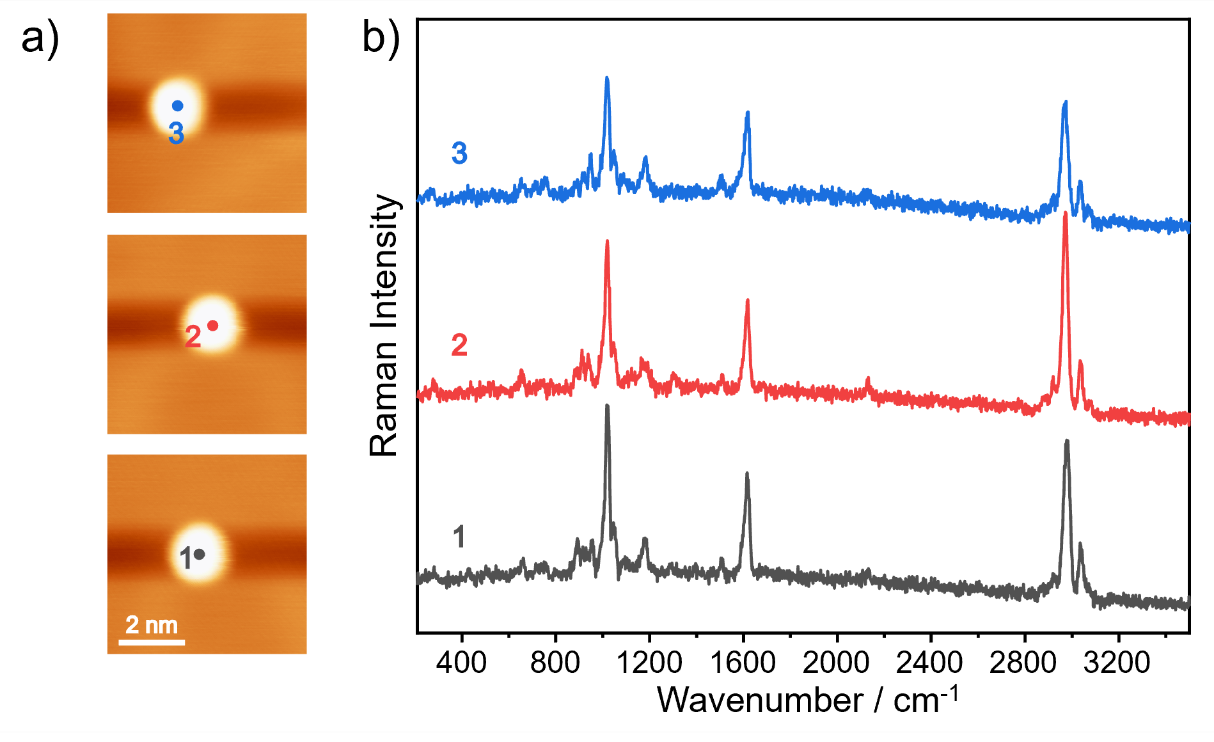


**Figure S4**: a) STM images (−1 V, 5 pA) of three single molecules (flat-lying) with the same height on a Au(111) terrace. b) Corresponding TERS spectra of these three molecules (−0.1 V, 1 nA, 20 s).


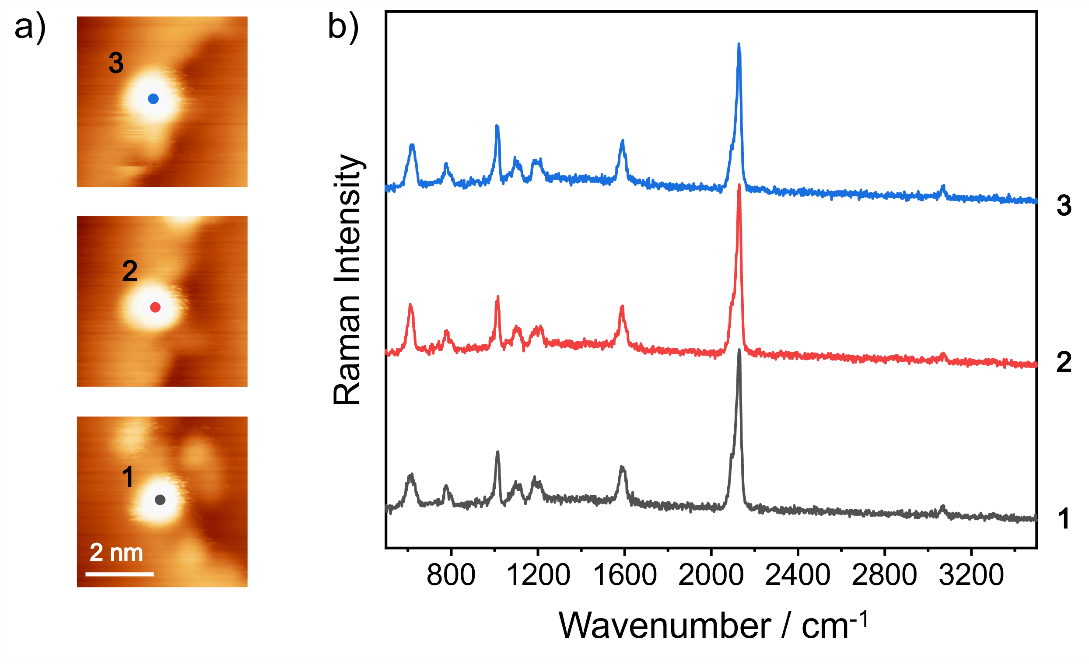


**Figure S5**. a) STM images (−1 V, 5 pA) of three single molecules (upright) at a step edge of the Au(111) surface. b) Corresponding TERS spectra of these three molecules (−0.1 V, 3 nA, 10 s).


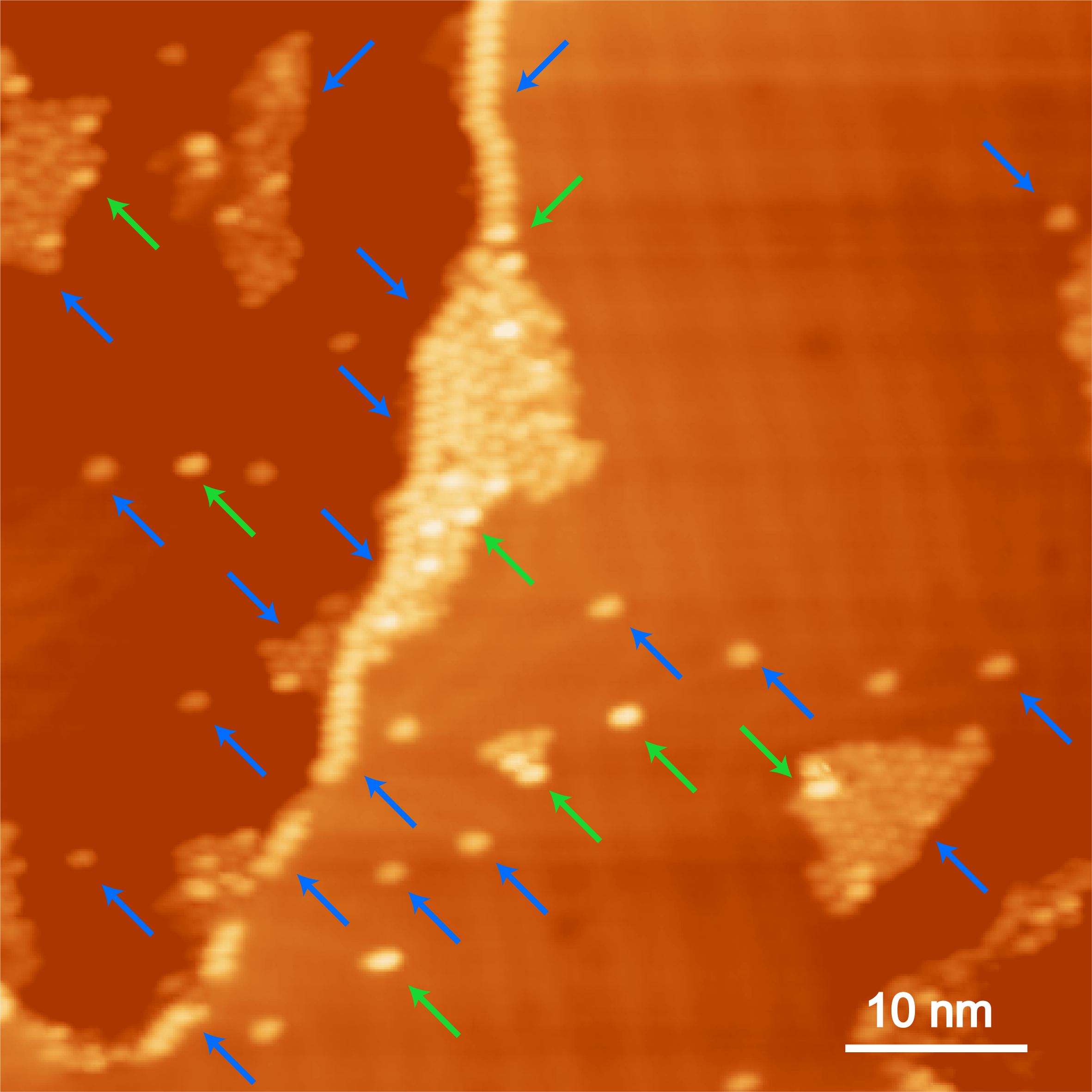


**Figure S6**: Large-scale STM image (60 × 60 nm^2^, −1 V, 5 pA) of alkyne-terminated tripod molecules on Au(111). Darker spots (blue arrows) are molecules that are standing upright. Brighter spots (green arrows) are flat-lying molecules.

# NMR data


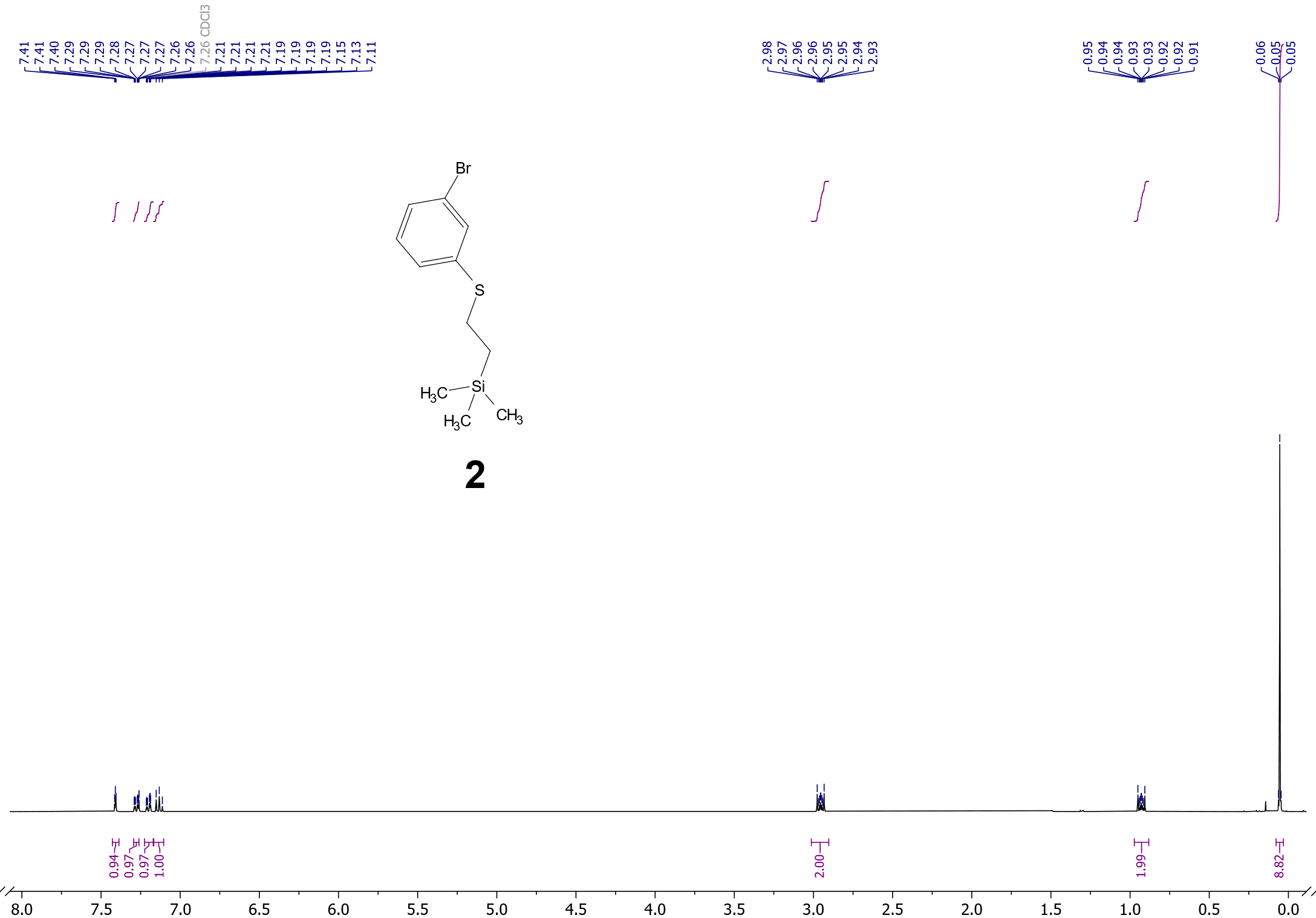


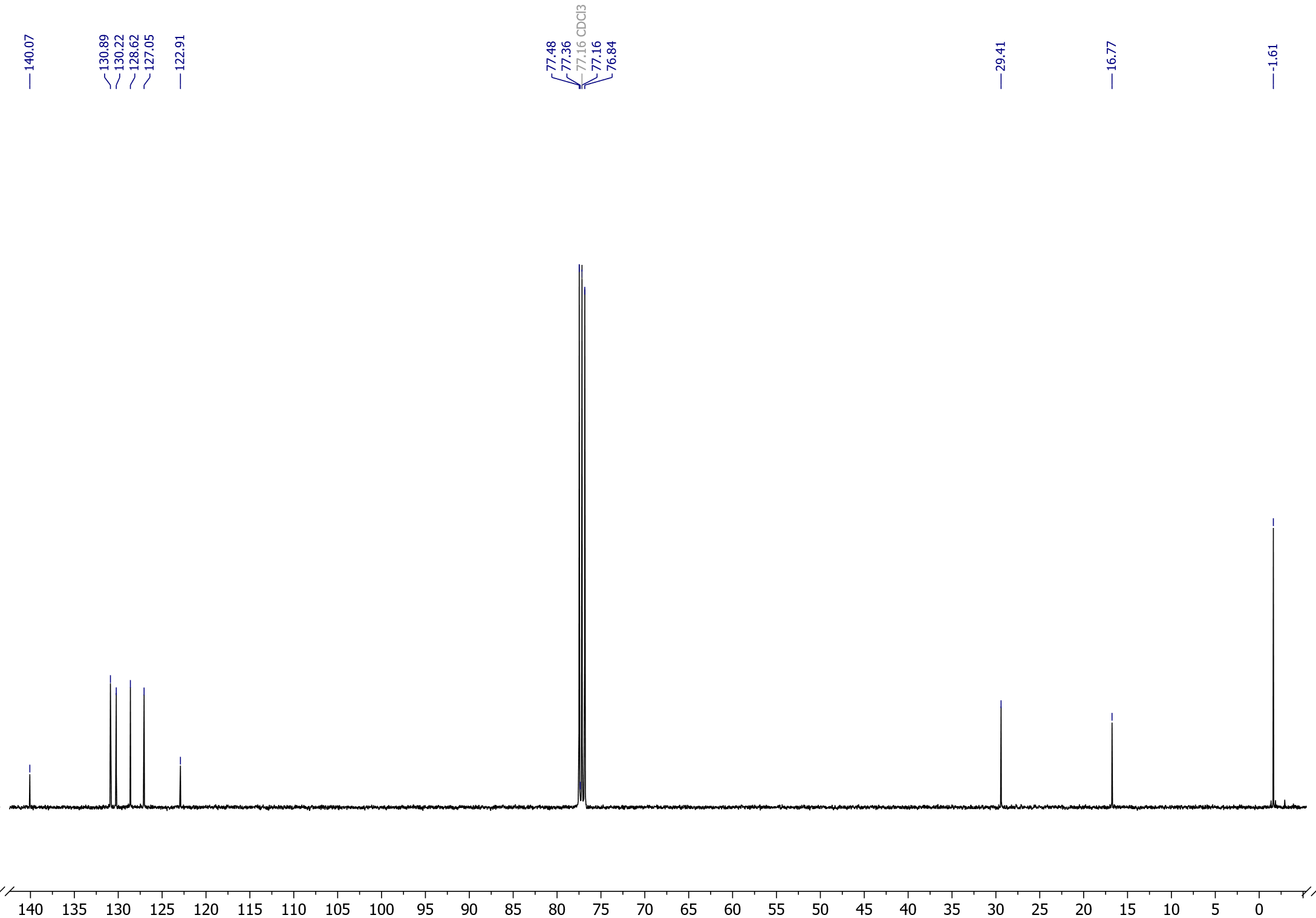


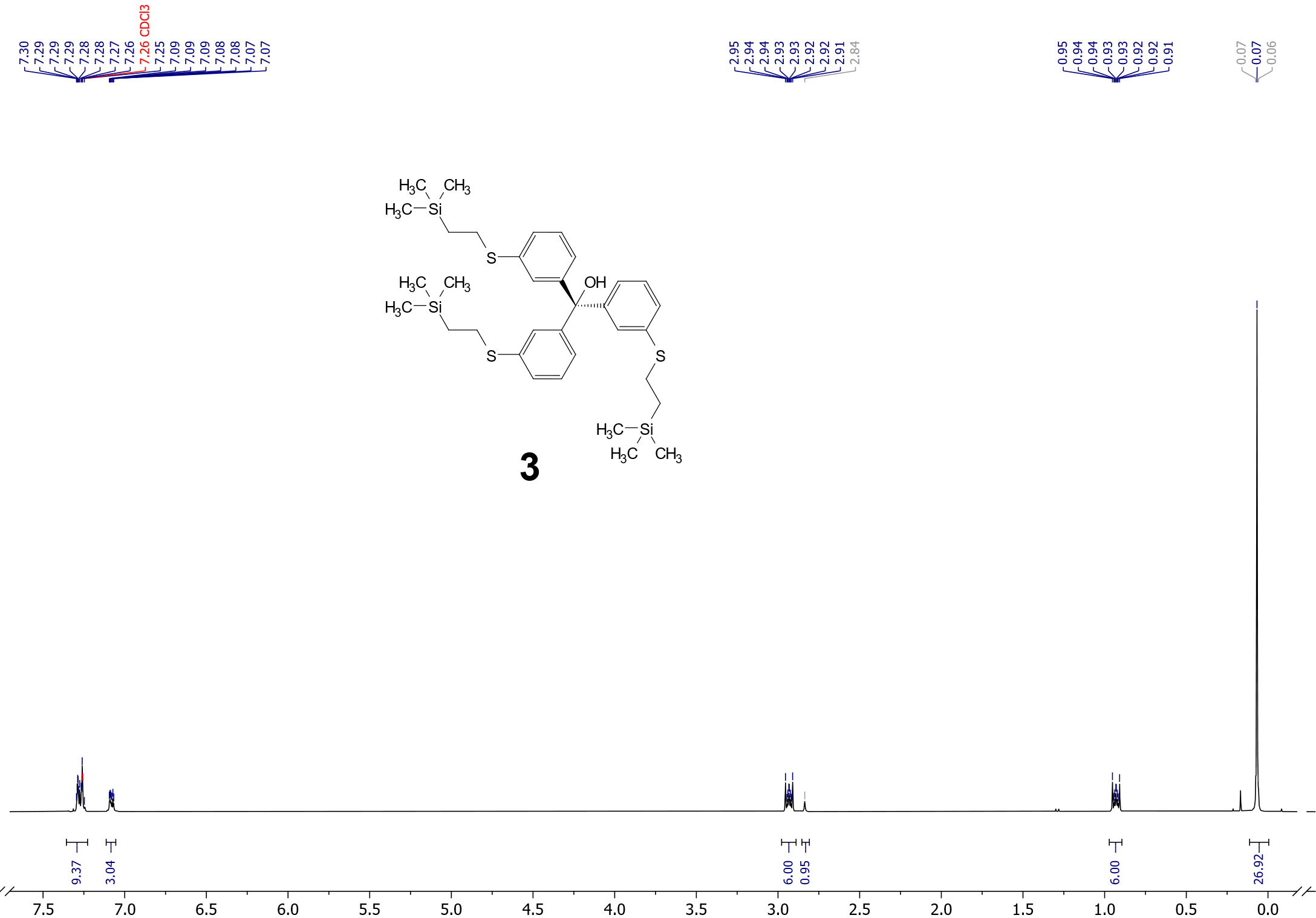


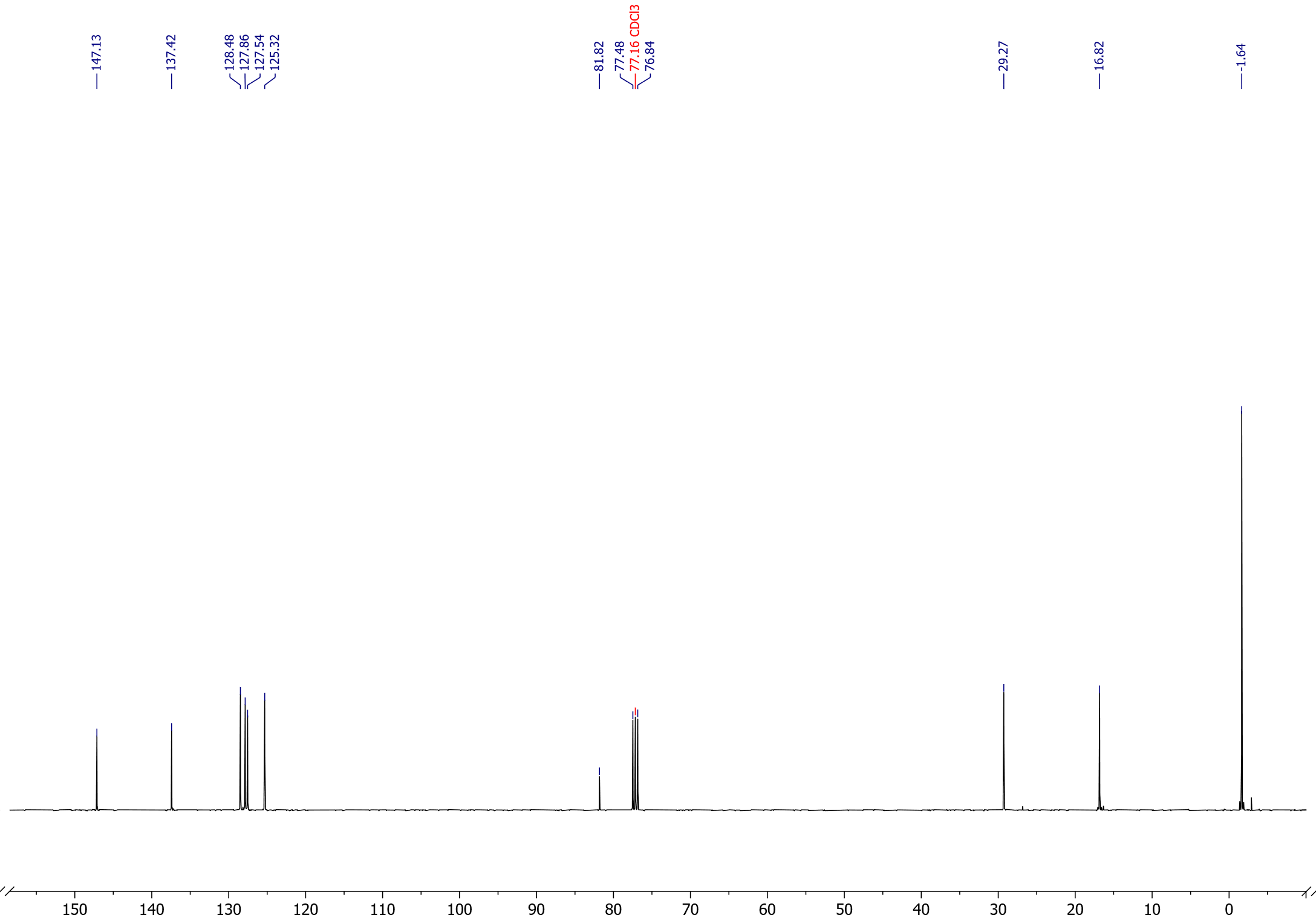


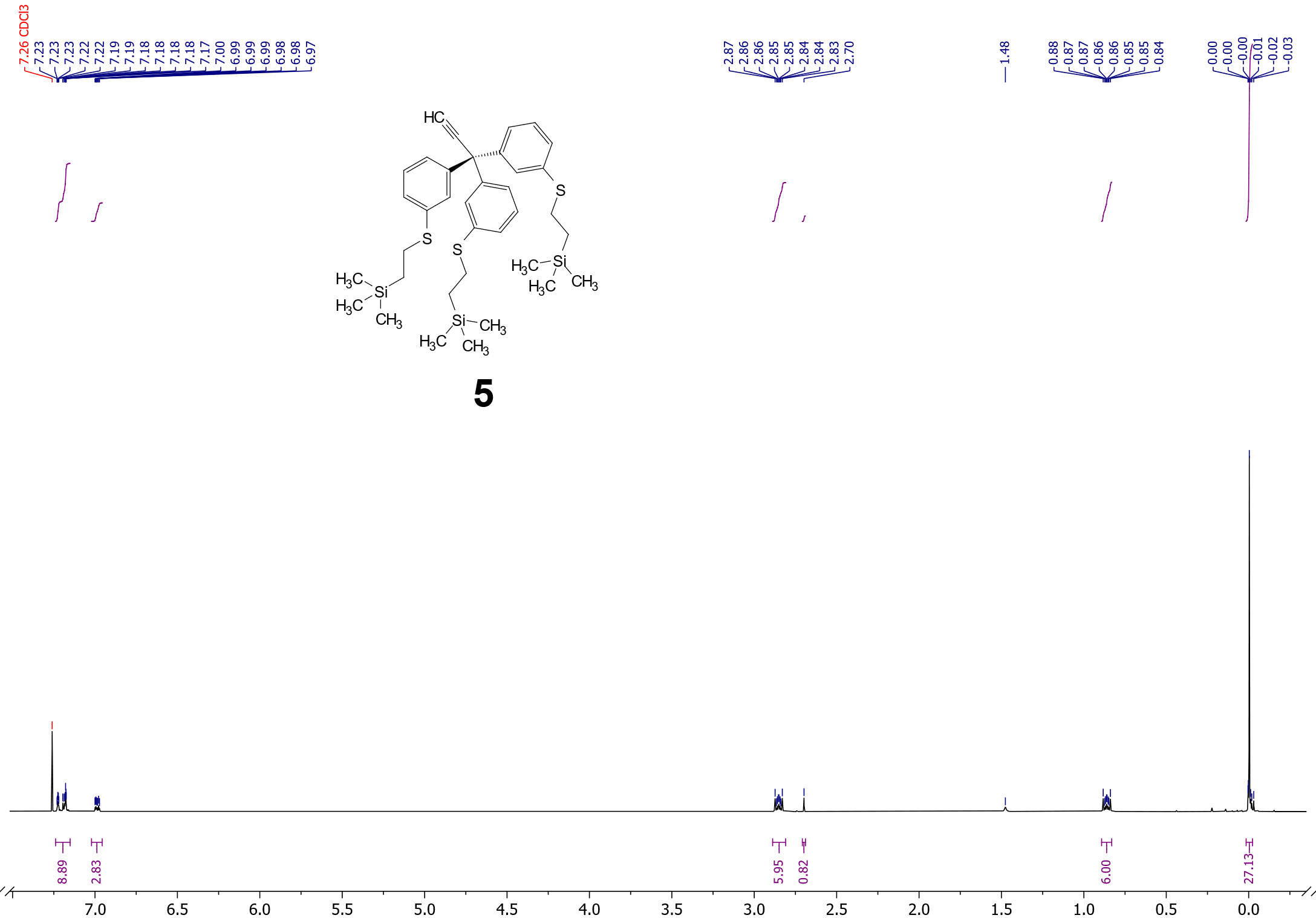


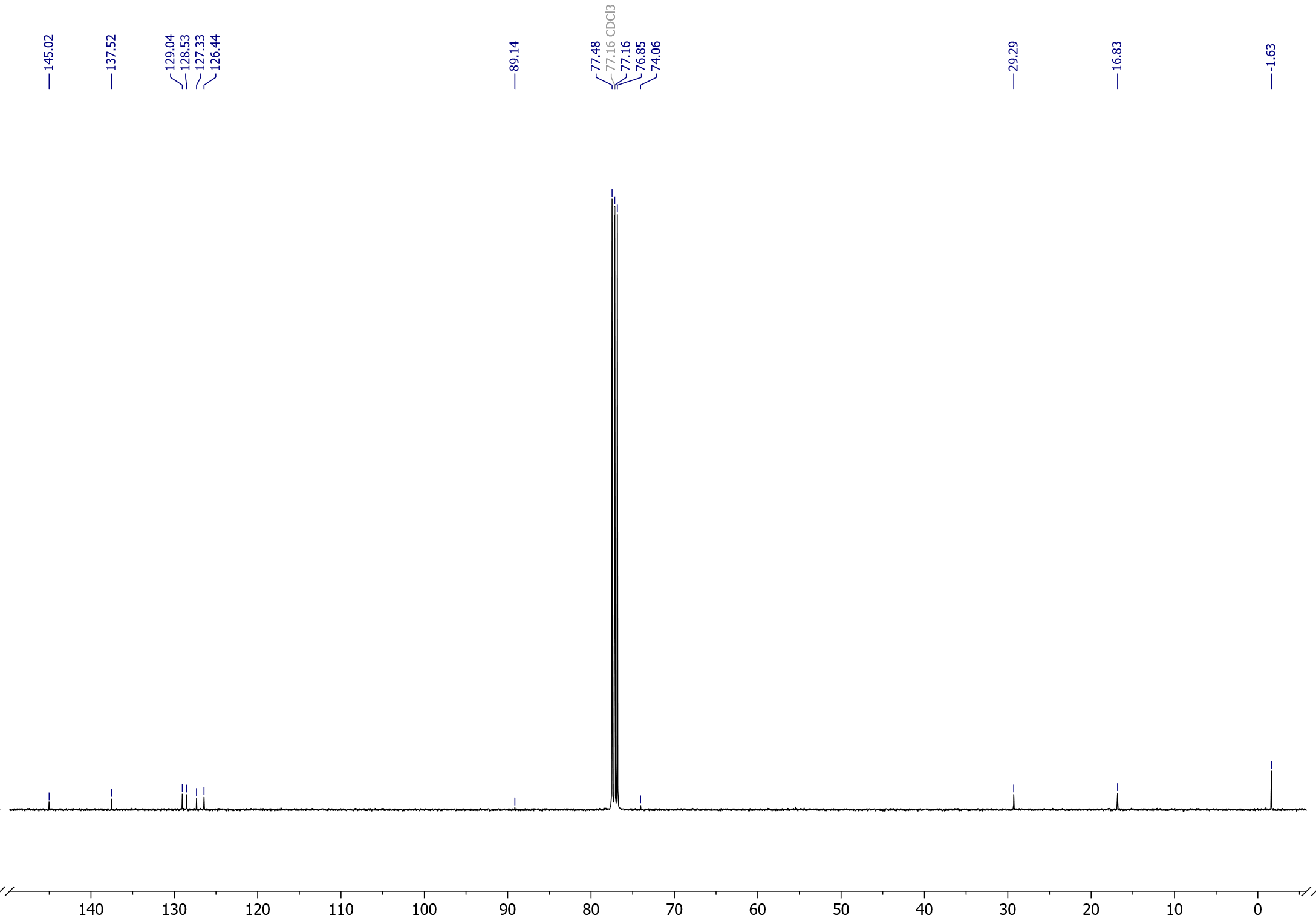


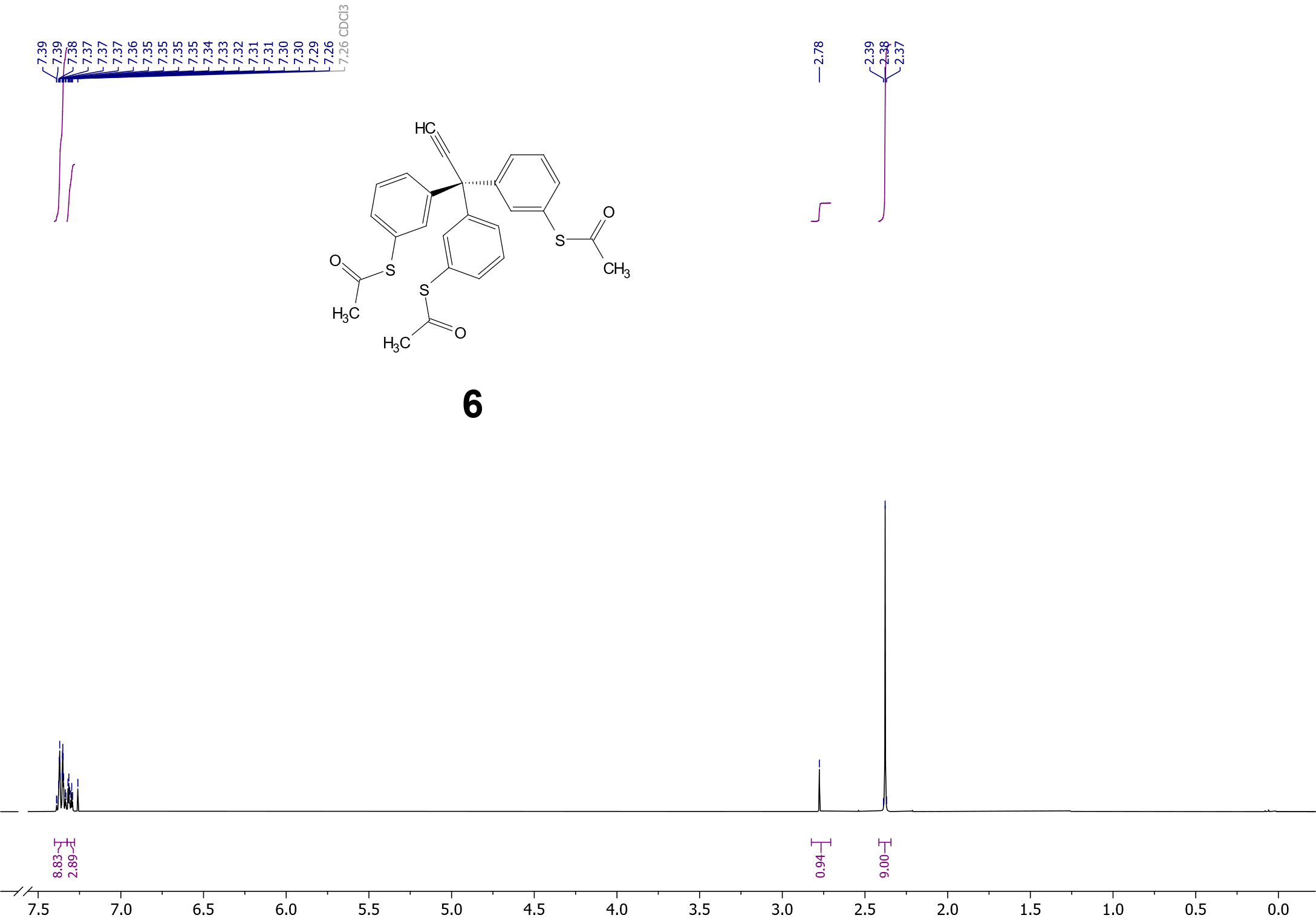


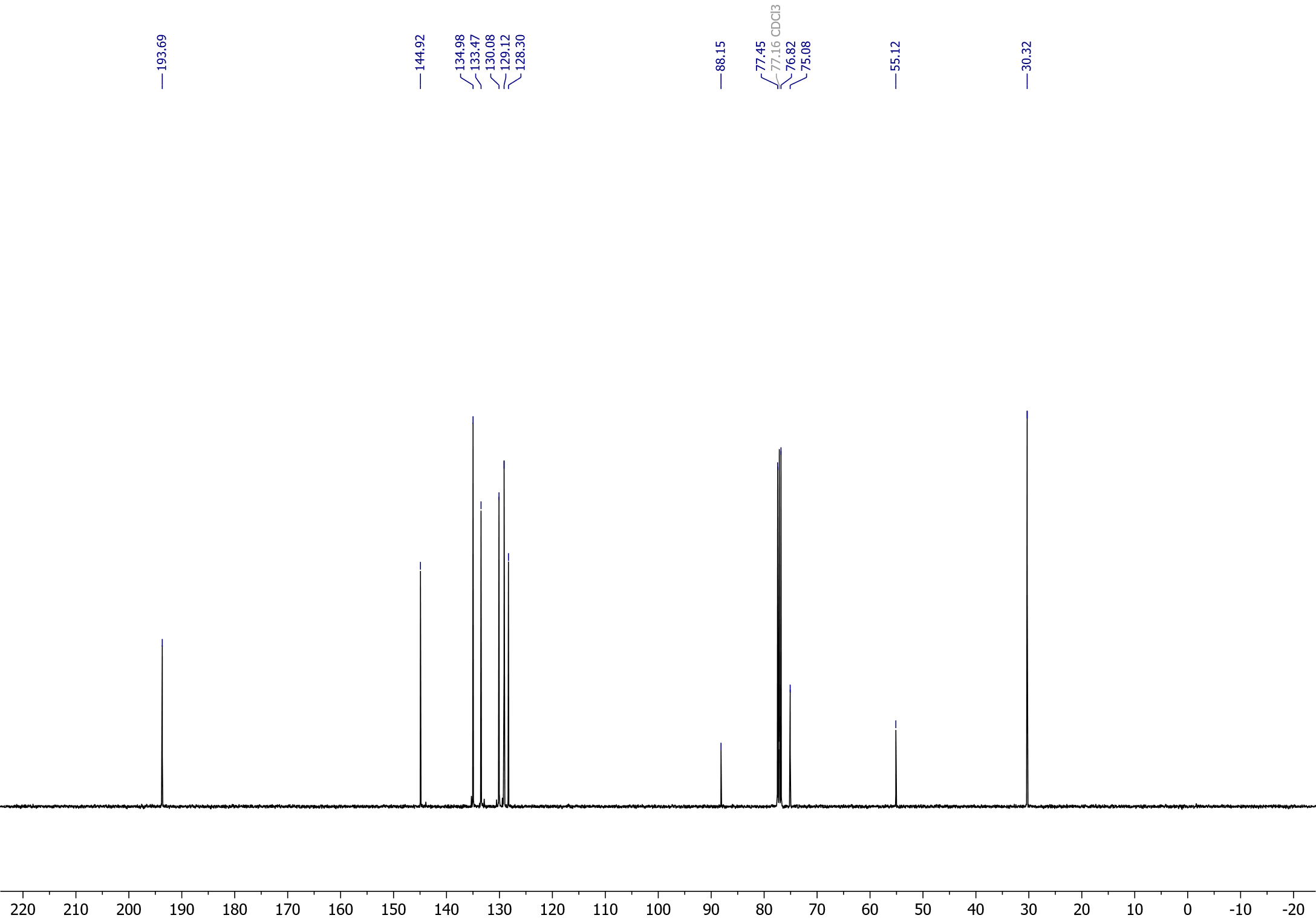


# Referemces

[1] Matulaitiene, I.; Kuodis, Z.; Matijoska, A.; Eicher-Lorka, O.; Niaura, G. SERS of the Positive Charge Bearing Pyridinium Ring Terminated Self-Assembled Monolayers: Structure and Bonding Spectral Markers. *J. Phys. Chem. C* **2015**, 119(47), 26481−26492. DOI: 10.1021/acs.jpcc.5b07687.
